# Enhanced mild-temperature photothermal therapy by pyroptosis-boosted ATP deprivation with biodegradable nanoformulation

**DOI:** 10.1186/s12951-023-01818-1

**Published:** 2023-02-23

**Authors:** Kaiyuan Liu, Li Zhang, Hengli Lu, Yingfei Wen, Bo Bi, Guocheng Wang, Yingying Jiang, Leli Zeng, Jing Zhao

**Affiliations:** 1 Scientific Research Center, The Seventh Affiliated Hospital of Sun Yat-Sen University, Shenzhen, 518107 People’s Republic of China; 2 School of Medicine, Tongji University, Shanghai, 200072 People’s Republic of China; 3 Research Center for Human Tissues and Organs Degeneration, Shenzhen Institute of Advanced Technology, Chinese Academy of Science, Shenzhen, 518055 Guangdong China; 4 Institute of Translational Medicine, Shanghai University, Shanghai, 200444 People’s Republic of China

**Keywords:** Pyroptosis, ATP deprivation, Mild-temperature PTT, Biodegradation, Osteosarcoma

## Abstract

**Background:**

Mild-temperature photothermal therapy (mild PTT) is a safe and promising tumor therapeutic modality by alleviating the damage of healthy tissues around the tumor due to high temperature. However, its therapeutic efficiency is easily restricted by heat shock proteins (HSPs). Thus, exploitation of innovative approaches of inhibiting HSPs to enhance mild PTT efficiency is crucial for the clinical application of PTT.

**Results:**

Herein, an innovative strategy is reported: pyroptosis-boosted mild PTT based on a Mn-gallate nanoformulation. The nanoformulation was constructed via the coordination of gallic acid (GA) and Mn^2+^. It shows an acid-activated degradation and releases the Mn^2+^ and GA for up-regulation of reactive oxygen species (ROS), mitochondrial dysfunction and pyroptosis, which can result in cellular ATP deprivation via both the inhibiton of ATP generation and incresed ATP efflux. The reduction of ATP and accumulation of ROS provide a powerful approach for inhibiting the expression of HSPs, which enables the nanoformulation-mediated mild PTT.

**Conclusions:**

Our in-vitro and in-vivo results demonstrate that this strategy of pyroptosis-assited PTT can achieve efficient mild PTT efficiency for osteosarcoma therapy.

**Graphical Abstract:**

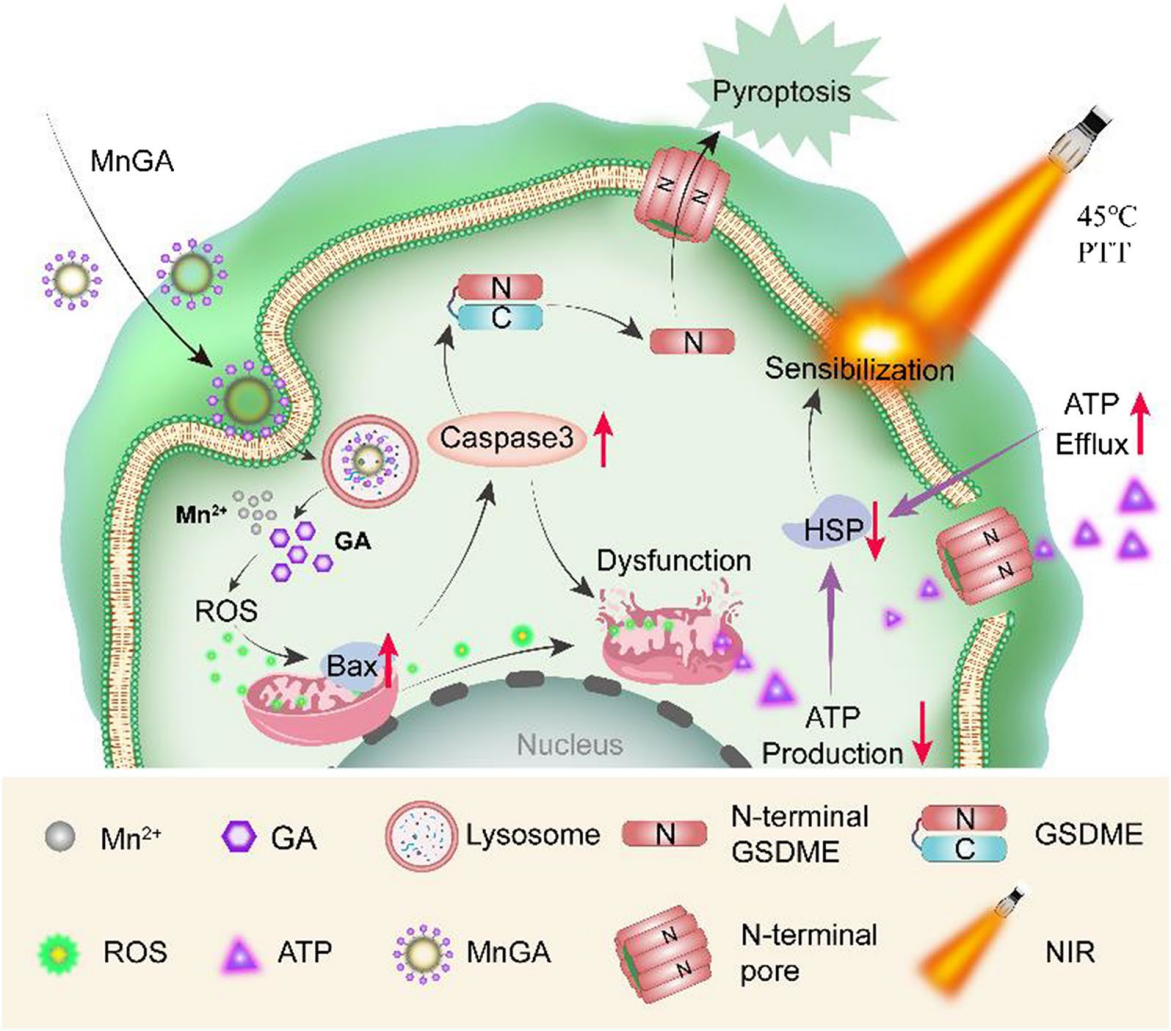

**Supplementary Information:**

The online version contains supplementary material available at 10.1186/s12951-023-01818-1.

## Introduction

Osteosarcoma (OS) is one of the most common primary malignant tumors occurring in bone, and chemotherapy is still the most common treatment. However, chemotherapy is prone to tumor recurrence, metastasis and multidrug resistance. Its therapeutic effect, especially for the improvement of the overall survival rate of patients with metastatic osteosarcoma, is still not satisfactory. Thus, finding novel treatment methods is imperative for effective clinical results. Photothermal therapy (PTT), the most effective treatment for cancer with the least level of invasiveness, has drawn great attention on account of its recent clinical and preclinical trials which have been very successful [1–3]. However, in order to completely eliminate tumor, PTT needs to generate hyperthermia (over 50 °C), which inevitably leads to adverse effects on surrounding normal tissues and causes the risk of recurrence or metastasis.[4, 5] Therefore, there is an urgent need to develop mild-temperature PTT (mild PTT) to lower hyperthermia offering an effective and safe therapeutic effect (< 45 °C) [6, 7].

Under mild temperature condition, the killing effect of PTT is far from sufficient due to the overexpressed heat shock proteins (HSPs) in cancer cells, [8, 9]which might rapidly restore thermal damage and may cause the thermoresistance of cancer cells. For example, such as the protein HSP70 and HSP90 are at a high expression in OS cells to participate protein folding and promote survival at unfavorable external conditions [10]. Some strategies of reducing the cancer cell’s thermoresistance mediated by HSPs have been exploited, such as the inhibition of HSPs expression or activity using gene silence or molecular inhibitors, along with the combination of mild PTT to boost cancer therapeutic effects [11–15]. In addition, mechanistic studies suggest that the involvement of HSPs in the tolerance process of cancer cells requires the participation of adenosine triphosphate (ATP) [16]. Importantly, the rapid synthesis and expression of HSPs in cancer cells under heat stress are intrinsically related to the cellular ATP levels [17]. These suggest that inhibiting the cellular ATP level may represent a promising approach to overcome the HSPs-dependent cancer thermoresistance during PTT.

Pyroptosis, a gasdermin-mediated lytic programmed cell death characterized by cell swelling, has emerged as a promising anti-tumor strategy. Encouragingly, some evidences show that the elicitation of apoptosis is accompanied by ATP efflux [18]. Thus, pyroptosis might provide a new direction for inhibiting HSPs. Recently, mitochondrial oxidative stress and dysfunction have been shown to be involved in reactive oxygen species (ROS)-mediated pyroptosis [19–21]. As mitochondria are the energy source of cells and produce ATP, their dysfunction might inhibit the cellular ATP supply. The increased ROS and less cellular ATP lever are also considered to contribute to the loss of function for HSPs [22, 23]. It is envisaged that the induction of pyroptosis through targeted modulation of mitochondria could be beneficial for effective mild PTT via the inhibition of HSPs.

**Fig. 1:**
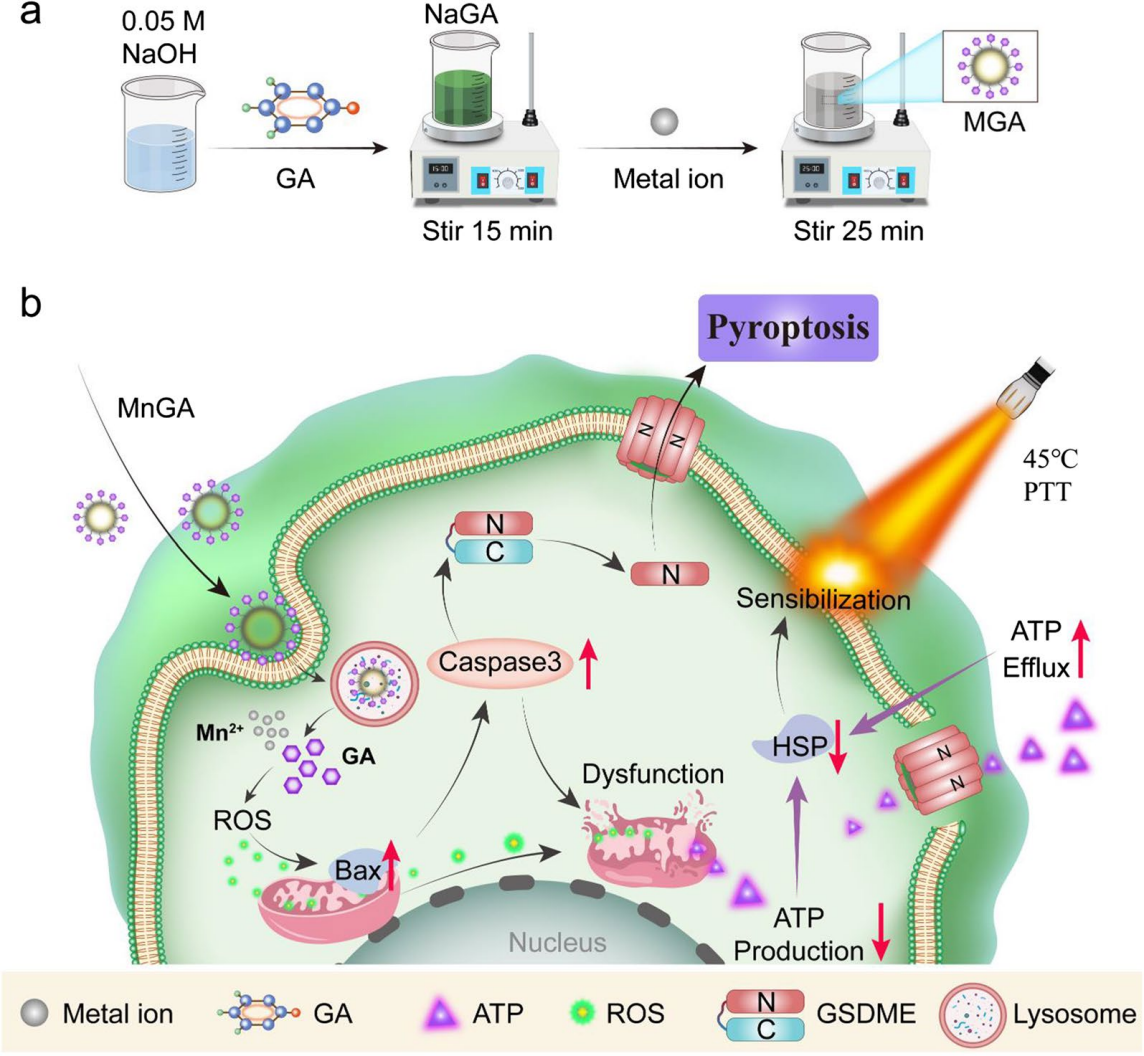
Schematic showing the synthetic process and mechanism of PTT-based antitumor effect induced by metal-gallate nanoparticles. **a** Schematic representation of the synthesis procedure of metal-gallate nanoparticles. **b** Rational antitumor design of mechanism for pyroptosis and enhanced mild-temperature PTT via downregulating HSPs through ATP exhausting

Herein we report a biodegradable nanoformulation strategy for mild temperature photothermal with potent potential for OS treatment. Briefly, Gallic acid (GA, 3,4,5-trihydroxybenzoic acid), a bioactive component of green tea with multiple biological functions including anti-inflammatory [24] and anti-cancer [25], was used. GA was nanoformulated by coordination with metal ions (Ca^2+^, Fe^2+^ and Mn^2+^) through a simple and mild synthetic method, as shown in Fig. 1a. The as-prepared metal-gallate nanoparticles were chemically stable at neutral pH conditions while became degradable at acidic solutious, showing potential merits of on-demand release of GA and metal ions at tumor sites and thus increasing cellular ROS level. Most importantly, the nanoformulated GA was found to be able to activate pyroptosis pathway of cancer cells beside apoptosis, and down-regulate the expression of HSPs via exhausting ATP and thus realizing enhanced mild PTT efficiency (Fig. 1b).

## Materials and methods

### Materials

Dulbecco’s Modified Eagle Medium (DMEM) culture medium with high glucose was purchased from Hyclone (American), and FBS was purchased from Gibco (American). gallic acid monohydrate, FeCl_3_, MnCl_2_·4H_2_O and CaCl_2_ were purchased from Aladdin (China). 5-FAM cadaverine was purchased from Fanbo Biochemicals Co. Ltd. The following antibodies were purchased commercially from Abcam (Britain): anti-HSP70 Ab, anti-HSP90 Ab, anti-Bcl-2 Ab, anti-Bax Ab, anti-cleaved N-terminal GSDME Ab, anti-Cleaved Caspase-3 Ab and anti-GAPDH Ab. DCFH-DA, Cell Counting Kit-8 (CCK-8), BCA Protein Quantitation Kit, Caspase 3 Activity Assay Kit, ATP Assay Kit and PI/calcein-AM were acquired from Beyotime Biological Co., Ltd. (Shanghai, China). The female Balb/c nude mice (4–6 weeks) were obtained from Charles River (Shanghai China).

### Synthesis of FeGA, CaGA and MnGA

Synthesis of Ca gallate (CaGC): 170 mg of gallic acid monohydrate was dissolved with 20 mL of 0.05 M NaOH solution, it was then added into 40 mL of 10 mM CaCl_2_ solution. Subsequently, this mixture was brought to a pH of 7.0 and stirred for 1 h at room temperature. After centrifugation and washing, CaGC nanoparticles were obtained. Synthesis of Fe gallate (FeGC): 170 mg of gallic acid monohydrate was dissolved with 20 mL of 0.05 M NaOH solution, it was then added into 40 mL of 10 mM FeCl_3_ solution. Subsequently, this mixture was brought to a pH of 7.0 and stirred for 1 h at room temperature. After centrifugation and washing by water and ethanol, FeGC nanoparticles were obtained. Synthesis of Mn gallate (MnGC): 170 mg of gallic acid monohydrate was dissolved with 20 mL of 0.05 M NaOH solution, it was then added into 40 mL of 10 mM MnCl_2_·H_2_O solution. Subsequently, the pH of this mixture was adjusted to 7.0 and stirred for 1 h at room temperature. After centrifugation and washing, MnGC nanoparticles were obtained. For the celluar uptake experiment, 5-FAM-labeled MnGA was synthesized by mixing MnGA and 5-FAM cadaverine and stirring for 24 h.

### Characterization of metal-gallate nanoparticles

Transmission electron microscopy (TEM, JEM-2100F) was used to examine the morphology, and energy dispersive X-ray spectroscopy (XPS, Thermo Scientific K-Alpha) was used to analyze the element content. A (Rigaku Ultima IV) X-ray diffractometer is used to obtain XRD patterns of FeGC, MnGC, and CaGC, while the UV–vis-NIR spectra were measured by (UV-1900i, SHIMADZU) spectrophotometers. Inductively coupled plasma-optical emission spectrometry (ICP-OES, Agilent 700 Series, Agilent Technologies, US) was used for the quantitative elemental analysis. Fourier transform infrared spectroscopy (FTIR) spectra were collected between 4000 and 400 cm^−1^ on a Nicolet 7000-C spectrometer. With the Micromeritics ASAP 2460 surface area analyzer, the specific surface areas of the Brunauer–Emmett–Teller (BET) compounds were determined. The particle sizes and zeta potential were detected by Zetasizer Nano Series (Malvern, USA).

### In vitro degradation

10 mg of metal-gallate nanoparticles was dissolved in 10 mL PBS (Ca^2+^ and Mg^2+^ free). After different incubation time at 37 °C, 100 μL supernatant was collected via centrifugation and inductively coupled plasma-optical emission spectrometry (ICP) was used to analyze the samples. The degradation rate was evaluated by cumulatively released metal ions (Fe^3+^, Mn^2+^ and Ca^2+^) in the supernatant.

### Cell culture, cytotoxicity and cellular uptake in vitro

The MG-63 cells were cultured in high-glucose DMEM containing 10% fetal bovine serum (FBS), 1% penicillin, and 1% streptomycin. A 5% CO_2_ atmosphere was used to incubate the cells at 37 °C. For staining viable and dead cells, Calcein-AM/propidium iodide staining reagents were used. Following multiple interventions, the media was replaced by 0.2 μM Calcein-AM/propidium iodide for 30 min coincubation. The Calcein-AM/propidium iodide solution was replaced by fresh media for 20 min at 37 °C. Fluorescence microscopy was used to observe the cells. In order to study MnGA uptake by MG-63 cells, 5-FAM-labeled MnGA was used in a fluorescence microscope. In a 12-well plate, 1 × 10^5^ MG-63 cells were seeded for attachment overnight. Each well was then injected with 5-FAM-labeled MnGA. Cells were washed 3 times in PBS after being incubated for 2 and 4 h, then stained with 4′,6-diamidino-2-phenylindole (DAPI) and washed again for 3 times. Lastly, confocal microscope was used to examine the cells.

### Wound healing, transwell invasion and colony formation assay

Colony formation assays were conducted as follows. To begin, 6-cm culture dishes were sown with MG-63 cell lines at the density of 1000 cells per plate and cocultured with DMEM containing GA and metal-gallate nanoparticles (5 μg/mL). A 14-day incubation at 37 °C with 5% CO_2_ was followed by a fixative step involving 4% paraformaldehyde for 20 min, followed by a crystal violet step lasting 15 min. Afterwards, the number of colonies with more than 50 cells were counted. Scratch wound assays were used to analyze cell migration. The 6-well plate was scratched vertically with a sterile pipetting tip and a cell monolayer adhered to its wall using 1 mL of sterile pipetting tip. Following this, the plate was washed three times with PBS to remove the detached cells. After that culture medium was replaced by serum-free DMEM medium containing GA as well as metal-gallate nanoparticles (10 μg/mL). Wound healing processes were recorded by a reverse phase microscope at 0, 24 and 48 h, and the wound closure ratios were estimated.

A matrigel-coated transwell chamber (Corning-Costar, 8-µm pore size) was used for in vitro assessment of invasion using transwell assays. Briefly, an upper chamber was seeded with 200 µL of cell suspension containing 5 × 10^4^ MG-63 cells, and 600 µL of DMEM containing GA and metal-gallate nanoparticles (10 μg/mL) was filled to the bottom chamber. Incubation at 37 °C with 5% CO_2_ for 24 h was followed by fixation with 4% paraformaldehyde for 20 min and staining with 1% crystal violet solution for 15 min to obtain migration cells. An inverted microscope was used to count cells in 10 random fields.

### Assessment of cytotoxicity assessment and antitumor effect in vitro with PTT

Photothermal therapy was performed using a near-infrared (NIR) laser device (808 nm, Shanghai Connect Fiber Optics Company) and a NIR thermal imaging camera (FLIRTM A325SC camera). A 96-well plate was seeded with MG-63 cells (1 × 10^4^ per well) and allowed to grow overnight for attachment. This was followed by addition of different concentrations of metal-gallate nanoparticles (0, 25, 50, 100, 200 μg/mL) to DMEM medium with or without NIR irradiation at 1.75 W/cm^2^ power density for 10 min. After incubation for 4 h, all the cells were removed from the medium. Each well was then filled with 10 μL of CCK8 solution and 100 μL of fresh medium and incubated for 2 h at 37 °C before being tested the absorbance at 450 nm.

### In vitro measurement of apoptosis and pyroptosis

To quantify the apoptotic cell population, the Apoptosis Detection Annexin kit was employed. Briefly, on 6-well plates, MG-63 cells (5 × 10^5^) were seeded and incubated overnight to ensure attachment. Then GA and metal-gallate nanoparticles were added into the medium for 6 h, then cells were harvested and collected by centrifugation. Afterwards, the cells were resuspended in 100 µL binding buffer and incubated with 5 µL Annexin V and 5 µL PI for 10 min at room temperature and analyzed by using a flow cytometer as directed by the manufacturer’s protocols. Bright field images, western blot (WB) and Caspase 3 activity assay were used for detection of pyroptosis after treatment with GA and metal-gallate nanoparticles. In brief, the protein levels related to apoptosis and pyroptosis (Bax, Bcl2, cleaved caspase-3, N-terminal GSDME and GAPDH) were measured using the western blot analysis. BCA Protein Quantitation Kit was used to determine the protein concentration in the total cellular proteins extracted using RIPA/PMSF 100:1 lysis buffer. According to the manufacturer’s protocol, the caspase-3 activity test was performed using the Caspase 3 Activity Assay Kit.

### DCFH-DA assay and JC-1 assay for cellular mitochondrial membrane change analysis in vitro

As a fluorescence probe, DCFH-DA was used to identify the intracellular ROS level, and CLSM was used to record the quantitative results. Briefly, a culture dish with glass bottom was seeded with MG-63 cells and incubated with DMEM containing GA and metal-gallate nanoparticles (50 μg/mL) for 2 h respectively. Afterwards, following three washing with PBS, incubation was performed at 37 °C for 20 min with 10 μm DCFH-DA in 1 mL of FBS-free DMEM. Cell samples were then washed three times with PBS and stained with 4′,6-diamidino-2-phenylindole (DAPI), followed by three more washings. Lastly, confocal microscopy was used to observe the cells. Likewise, MG-63 cells were also seeded in a dish having glass bottom at a density of 1 × 10^5^ cells per well and incubated for 24 h at 37 °C. Following this, fresh medium containing GA and metal-gallate nanoparticles (50 μg/mL) was added and the cells were incubated for another 2 h. After three PBS washes, all the cells were replaced with medium without FBS. A 30 min staining procedure with 10 μg/mL JC-1 was conducted at 37 °C. Lastly, fluorescence microscope was used to examine all the cells after washing.

### Expression of heat shock proteins (HSPs) and production of adenosine 5′-triphosphate (ATP)

A density of 2 × 10^5^ MG-63 cells per well was seeded in six-well plates and incubated at 37 °C for 24 h. The cells were then co-incubated with MnGA (20 μg/mL) at 37 °C or 43 °C overnight. Thereafter, the medium was collected for the test of extracellular ATP levels in accordance with the manufacturer’s protocol. Treated cells were used for intracellular ATP level detection in accordance with the ATP assay kit’s instructions. And cells were also collected for the analysis of heat shock proteins using the WB analysis with monoclonal anti-HSP 70 antibody and monoclonal anti-HSP90 antibody.

### Establishment of tumor bearing mice model and in vivo anti-tumor therapeutic effect

The female balb/c nude mice (4–6 weeks) were subcutaneously injected with MG-63 osteosarcoma cancer cells (2 × 10^6^ per mouse) in the right flank. As soon as the tumor volume reached about 50 mm^3^, the nude mice were divided randomly into four groups (n = 4): (1) PBS (100 μL), (2) NIR group (808 nm, 1.5W/cm^2^), (3) MnGA (2 mg/mL, 100 μl), (4) MnGA plus NIR. After intravenous injection for 8 h, mice in NIR group and MnGA plus NIR were irradiated for 10 min with an 808-nm laser (1.5 W/cm^2^). Every two days, we measured and recorded the tumor volume and body weight. The tumor volume was calculated using the previous formula: $Tumor \,volume=\left(length \times {width}^{2}\right)/2$. For the staining of the tumors with hematoxylin and eosin and TUNEL, tumors were collected. Total protein of tumor tissues was extracted as previous studies reported, and the expression of Caspase-3, N-terminal GSDME and GAPDH was analyzed by western blotting.

### Analysis of statistical data

Means ± standard deviation were used to represent continuous variables. SPSS statistical software version 25 (IBM Corp, Armonk, NY) was used to calculate multiple comparisons among different groups using a one-way ANOVA. There were significant differences when the ***p < 0.001, **p < 0.01, or *p < 0.05 represented significant difference.

## Results and discussion

### Preparation and characterization of FeGA, CaGA and MnGA metal-gallate nanoparticles

**Fig. 2:**
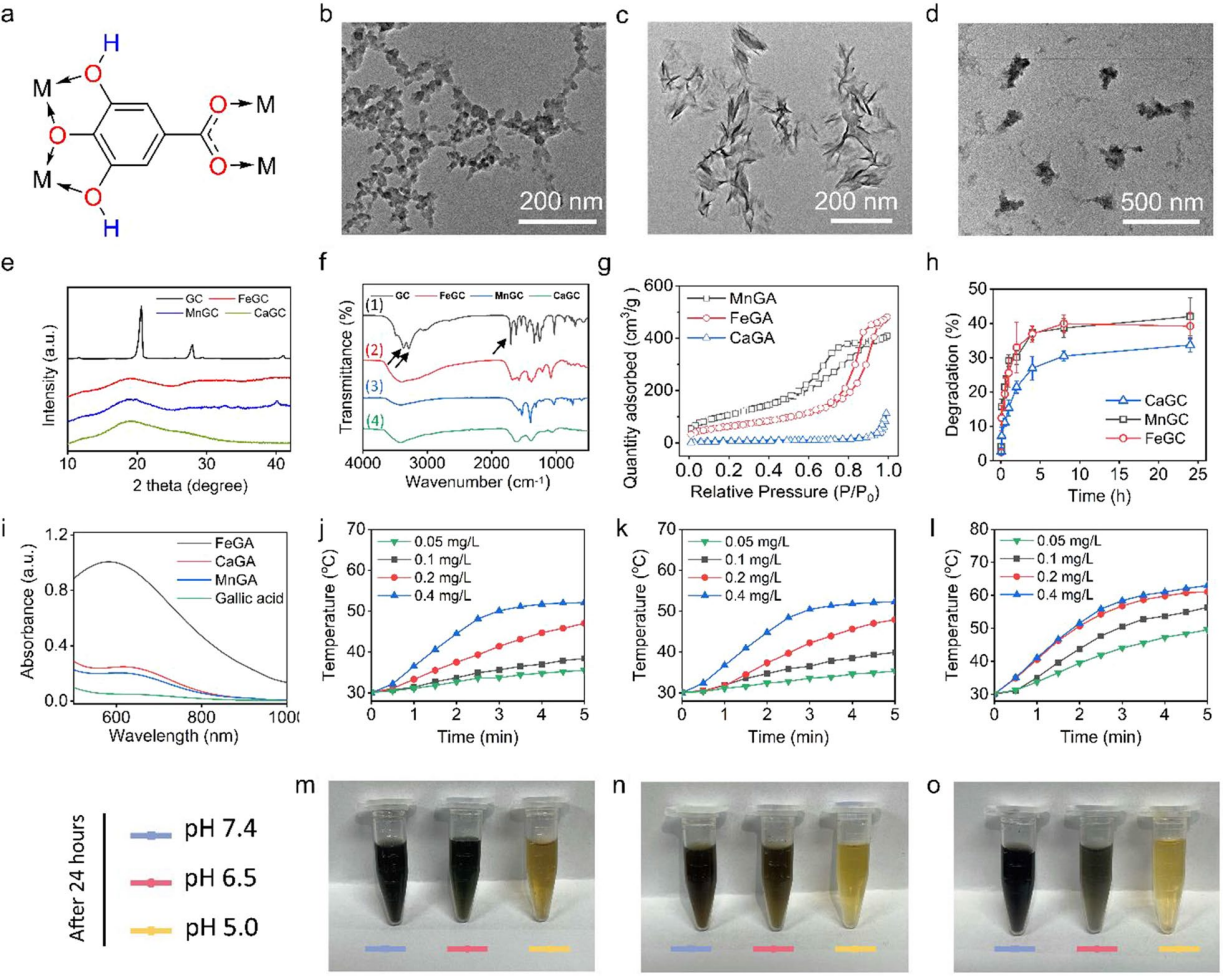
Characterizations of metal-gallate nanoparticles. **a** Schematic structure of coordination between gallic acid and metal ions (M represent metal ions). **b**–**d** TEM images of CaGA, MnGA and FeGA nanoparticles. **e** XRD patterns of FeGA, MnGA and CaGA nanoparticles. **f** FTIR curves of gallic acid, FeGA, MnGA and CaGA nanoparticles. **g** Specific surface area of FeGA, MnGA and CaGA nanoparticles. **h** Degradations of FeGA, MnGA and CaGA nanoparticles in PBS at pH 7.4. **i** Vis–NIR spectra of GA, FeGA, MnGA and CaGA nanoparticles. **j**–**l** Temperature changes of CaGA, MnGA and FeGA nanoparticles at various concentrations (0.05, 0.1, 0.2, 0.4 mg/L) after 808 nm NIR laser irradiation. **m**–**o** Photographs of CaGA, MnGA and FeGA in PBS with pH 7.4, 6.5 and 5.0

As shown in Fig. 2a, GA has phenolic hydroxyl and carboxyl groups which can be served as organic ligands to covalently bond to metal atoms. In this study, metal-gallate nanoparticles were synthesized through a simple and mild synthetic method without any nanocarriers, which could avoid the toxicity and immunogenicity brought by the nanocarriers [26]. Three different types of metal cations, Ca (II), Fe (III) and Mn (II) were used for the nanoformulation of gallic acid, and resulting the formation of three different gallic acid-based nanoparticles. As shown in Fig. 2b–d, the as-prepared CaGA, FeGA and MnGA showed different morphologies and particle sizes. XRD results revealed that CaGA and FeGA nanoparticles were amorphous, while MnGA nanoparticles were low-crystalline with weak diffraction peaks at 32.6, 34.5 and 40 degrees (Fig. 2e). DLS analysis showed the average size of FeGA, MnGA and CaGA were 105.7, 164.2 and 295.3 nm, respectively (Additional file 1: Figure S1). Additional file 1: Figure S2 illustrates that each type of metal-gallate nanoparticles had a negative zeta potential due to the existence of carboxyl and hydroxyl groups (− 2.9 for FeGA, − 8.1 for MnGA and − 4.5 for CaGA, respectively). FTIR spectra revealed that after the coordination with metal ions, the stretching vibration of phenolic group at 3200–3400 cm^−1^ and carboxyl group at 1684 cm^−1^ (indicated by black arrows) disappeared, indicating that H of GA was replaced by metal ions (Fig. 2f) [27, 28]. As shown in Fig. 2g, the MnGA had a relatively higher specific surface area (374.8 m^2^/g). Besides, the existence of metal element in each metal-gallate nanoparticles was further validated through EDS as depicted in Additional file 1: Figure S3. The degradation rates of metal-gallate nanoparticles in PBS of pH at 7.4 were around 35–40% (Fig. 2h), which were ascribed to the breakdown of the reversible coordinate bonds between GA and metal ions in the presence of hydrogen ion. As shown in Additional file 1: Figure S4, the metal-gallate nanoparticles gradually degraded with the pH decrement, and the thorough degradation was achieved when pH reached 5.0, which showed the characteristic of acid-activated degradation. Additional file 1: Figure S5 showed that as-prepared metal-gallate nanoparticles exhibited high dispersity in deionized water and formed dark green or dark violet solutions, revealing the absorption of visible light and potential photothermal performance of those materials. As proved by UV–Vis-NIR spectra (Fig. 2i), there was an absorption peak at 580 nm for FeGA, and 615 nm for CaGA and MnGA. As shown in Additional file 1: Figure S6, the mass extinction coefficient of the NPs at 808 nm calculated from the Lambert–Beer law was 4.0 L g^−1^ cm^−1^ (FeGA), 3.25 L g^−1^ cm^−1^ (CaGA) and 3.01 L g^−1^ cm^−1^ (MnGA) respectively. Further, the photothermal-conversion efficiency (η) of the metal-gallate nanoparticles was calculated according to the previous formula [29]. Based on Additional file 1: Figure S7, the η of FeGA, MnGA and CaGA were 28.2%, 20.4% and 18.9%, respectively. Then the photothermal performance of metal-gallate nanoparticles was evaluated by irradiation with a NIR laser of 808 nm and a power density of 2 W cm^−2^. As shown in Fig. 2j–l as well as Additional file 1: Figure S8, FeGA exhibited increased photothermal conversion efficiency than CaGA and MnGA on account of its relatively stronger absorption of 808 nm light. pH-triggered degradations of metal-gallate nanoparticles were shown in Fig. 2m–o, that the color of the solution became lighter as the pH decreased due to the reversible coordinate bonds between GA and metal ions in the presence of massive H^+^. In addition, size and NIR absorption are proved to be relatively stable within 24 h in DMEM, which guarantees the stability of nanoparticles in vivo to perform PTT (Additional file 1: Figure S9 and S10). Although Fe ions and gallic acid have been jointly used to construct therapeutic nanoparticles for anti-cancer purposes, the synthesis of these nanoparticles commonly involves the use of carriers, like polyvinylpyrrolidone (PVP), BSA and mesoporous silica [30–32]. The multicomponent design inevitably led to side effects and immunogenicity.

### In vitro evaluation of anticancer of GA and metal-gallate nanoparticles

**Fig. 3:**
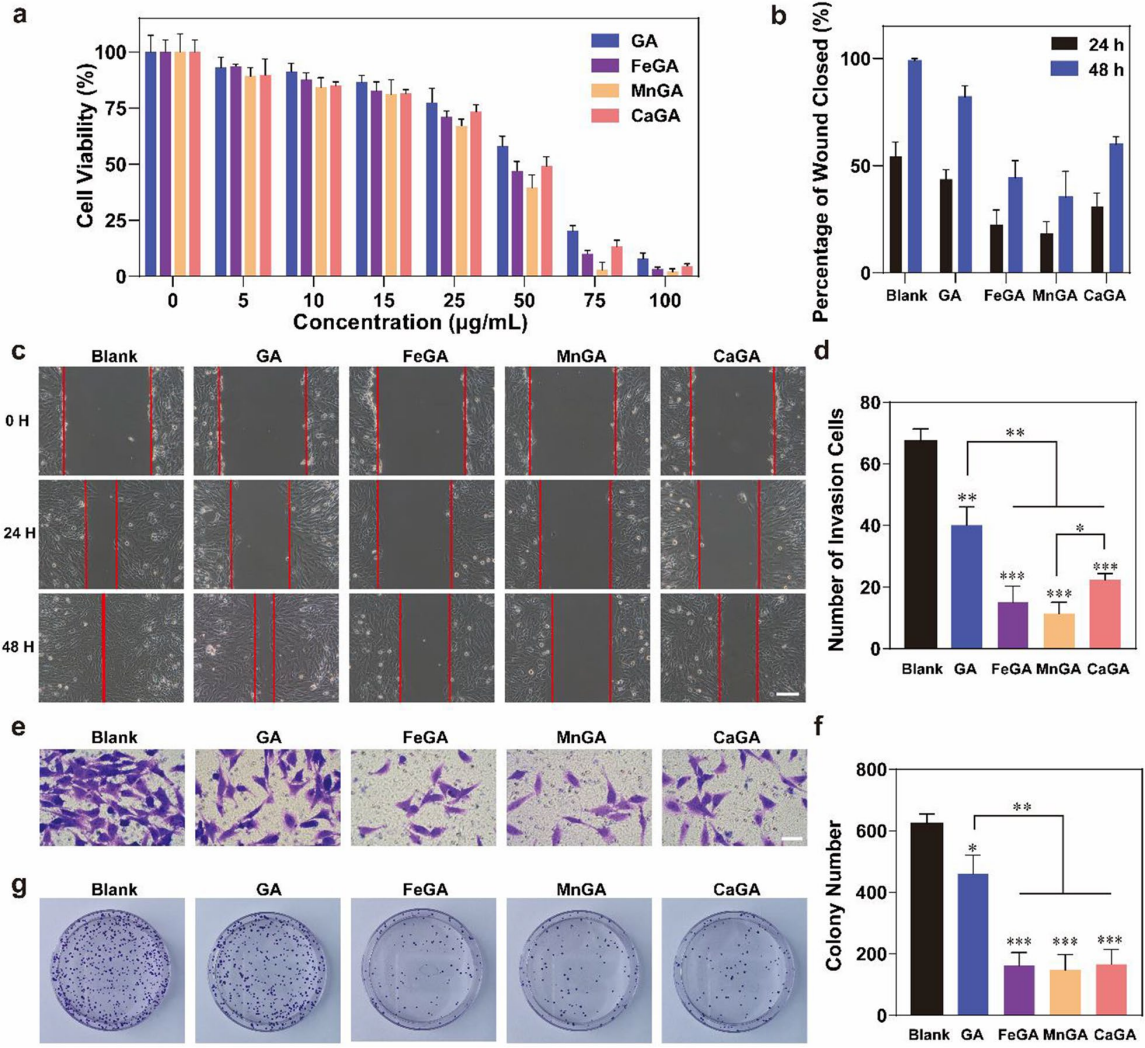
In vitro anticancer evaluation of GA and metal-gallate nanoparticles. **a** The cytotoxic effect of GA and metal-gallate nanoparticles measured by CCK8. **b**, **c** Effect of GA and metal-gallate nanoparticles on cell migration which was measured via wound healing assays. **d**, **e** The effect of GA and metal-gallate nanoparticles on cell invasion as measured by transwell assays. **f**, **g** Effect of GA and metal-gallate nanoparticles on cell proliferation measured by the number of colonies. *p < 0.05, **p < 0.01, ***p < 0.001. The scale bar is **c** 200 μm, **e** 100 μm

To determine the cytotoxicity of GA and metal-gallate nanoparticles against cancer cells, different concentrations of GA and metal-gallate nanoparticles were applied to MG-63 cells for 24 h, and their viabilities were evaluated by CCK-8 assays. As shown in Fig. 3a, compared with GA, metal-gallate nanoparticles had an obviously enhanced cytotoxicity against MG-63 cells when the concentration exceeded 25 μg/ml due to the biological activity of bound metal ions. Among the three types of metal-gallate nanoparticles, MnGA exhibited the highest degree of cytotoxicity, then by FeGA, while CaGA showed the lowest cytotoxicity against the MG-63 cell. Fe and Mn are known to catalyze the Fenton reaction in cancer cells and lead to excessive ROS production and organelle damage [33]. It should be noted that Mn(II) ions have a higher ability to catalyze Fenton reaction than Fe(III) ions [34, 35], which may explain the highest cytotoxicity of MnGA nanoparticles.

A wound healing assays as well as transwell assays were also used to study the effects of GA and metal-gallate nanoparticles on tumor cell migration and invasion in vitro. As shown in Fig. 3c and e, GA alone showed a certain degree of ability to inhibit tumor migration, however, nanoformulated GA with metal ions showed a much-enhanced anti-migration effect, which was more clearly evidenced by the quantitative data (Fig. 3b and d). Among the formulated GA nanoparticles, MnGA showed a better inhibitory effect against MG-63 cells compared with FeGA and CaGA. As depicted in Fig. 3f and g, the formulated metal-gallate nanoparticles had an obviously enhanced ability to reduce the clone formation compared to the free GA and no obvious difference in the inhibitory effects was found among the three metal-gallate nanoparticles. Considering the toxicity of all formulations at concentrations higher than 50 μg/mL, 5 μg/mL was chosen for colony formation assays, and 10 μg/mL for wound healing assays and transwell assays respectively. Collectively, the above results demonstrate that the nanoformulation of GA significantly enhanced the inhibition of proliferation of cells, migration and invasion of MG-63 osteosarcoma cell lines by GA, and point out the superiority of MnGA over the others. Although the MnGA nanoparticles show promising antitumor capacities, Fe ions have been prevailingly used to construct gallic acid-based nanoparticles for anticancer purposes, probably due to their superior photothermal properties (Fig. 2I) [31, 36]. To the best of our knowledge, no Mn- gallic acid nanomaterials have been so far reported.

### Intracellular ROS generation and pyroptosis induced by metal-gallate nanoparticles

**Fig. 4:**
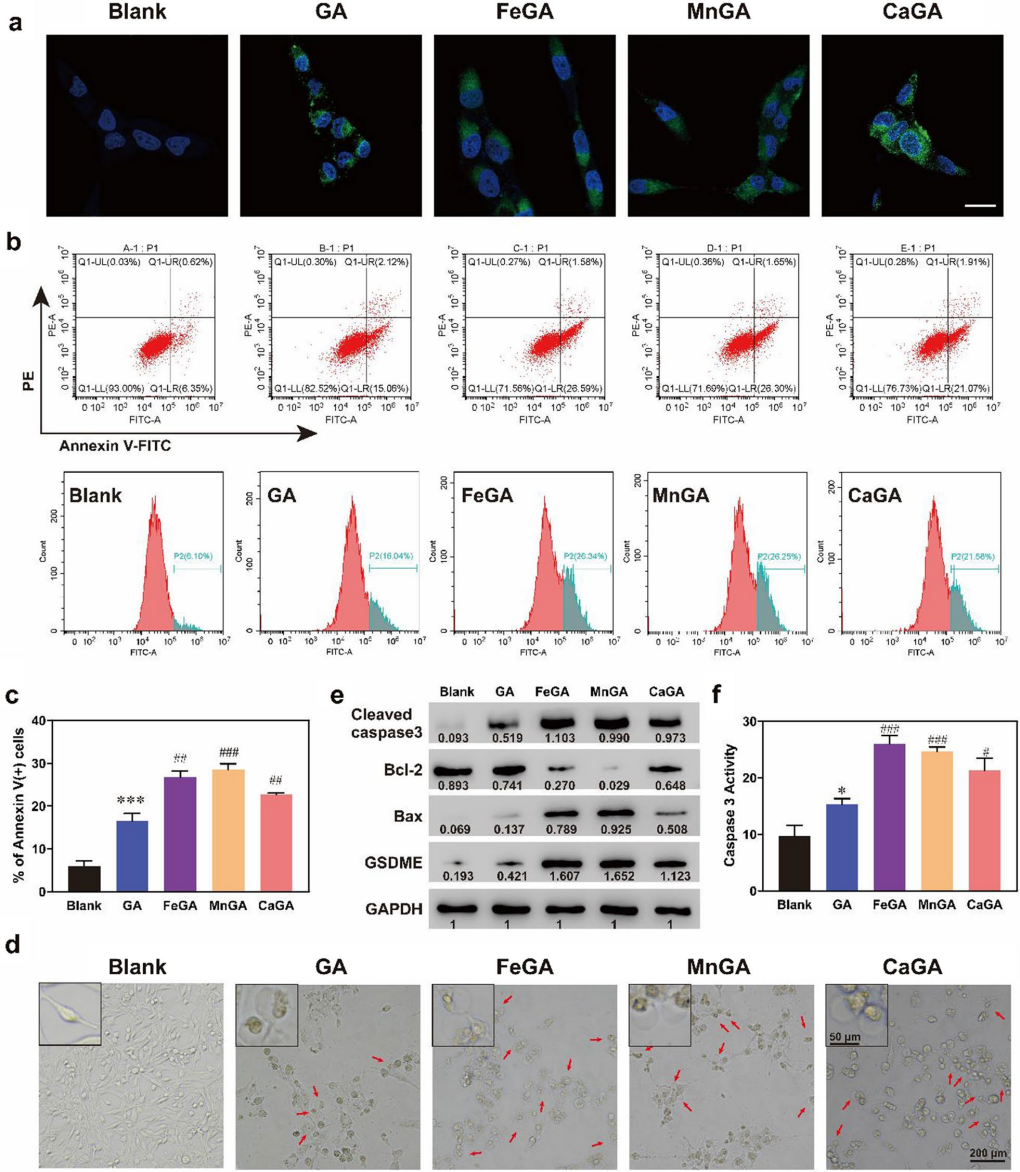
Intracellular ROS generation and pyroptosis induced by metal-gallate nanoparticles. **a** DA-DCFH fluorescence of MG-63 tumor cells exposed to PBS, GA, FeGA, MnGA and CaGA Nanoparticles. The scale bar is 20 μm. **b**, **c** MG-63 cells were cultured with PBS, GA, FeGA, MnGA and CaGA for 6 h, and flow cytometry was used to measure the apoptosis ratios for each group (Annexin V^+^ cells). **d** Bright field images of morphology of MG-63 cells after different treatments. **e** Representative western blots of N-terminal gasdermin E (N-GSDME), Bax, cleaved caspase-3 and Bcl-2 expressions in MG-63 cells treated with different formulations above. **f** Caspase-3 activity which was tested by Caspase 3 Activity Assay Kit in MG-63 cells treated with different formulations above. *p < 0.05, ***p < 0.001 (when compared with Blank group). ^#^*p* < 0.05, ^##^*p* < 0.01, ^###^*p* < 0.001(when compared with group of GA)

In this study, intracellular ROS production ability was detected in MG-63 cells using a ROS probe of 2,7-dichlorodihydrofluorescein diacetate (DA-DCFH). Figure 4a showed that GA induced slight ROS production, which was in accordance with previous studies [37]. However, FeGA, MnGA and CaGA groups induced more ROS in cells, as evidenced by the higher fluorescence intensity, compared to the GA group.

It was reported that excessive intracellular ROS could trigger mitochondrial permeability transition pore (mPTP) induction and exaggerate ROS production, which eventually leads to perceptible mitochondrial and cellular injury [38, 39]. Hence, the mitochondrial dysfunction of MG-63 osteosarcoma cells caused by metal-gallate nanoparticles evaluation was conducted by detecting the changes of their mitochondrial membrane potential (MMP) via 5,5′,6,6′-tetrachloro-1,1′,3,3′-tetraethylbenzimidazolyl carbocyanine-iodide (JC-1) fluorescent probe. As shown in Additional file 1: Figure S11, red fluorescence was intense in the PBS group, while green fluorescence was weak, indicating negligible MMP changes. After treatment with metal-gallate nanoparticles, the cells presented strong green monomeric fluorescence signals, representing mitochondrial dysfunctions. To confirm if the induced mitochondrial dysfunction could cause irreversible programmed cell death, flow cytometry was used for analysis of apoptosis. As can be seen from Fig. 4b, c, although GA effectively induced cell apoptosis, the formulated metal-gallate nanoparticles could significantly enhance the apoptosis of MG-63 cells. Among them, MnGA was superior to others. It is worth noting that the treated MG-63 cells did not present a typical morphology of the dead cells by apoptosis. As shown in Fig. 4d, the untreated cells kept uniform elongated morphology, while large amounts of pyroptotic cells were observed in GA and metal-gallate nanoparticles group, especially the metal-gallate nanoparticles group. Large bubbles blowing from plasma membrane were found in the cells treated with GA or metal-gallate nanoparticles (inset and red arrows in Fig. 4d), which is a typical morphological characteristic of cell pyroptosis. Recently, it was reported that iron-activated ROS could promote pyroptosis via a Bax-caspase3 pathway, and caspase-3 was a pyroptosis inducer through cleaving GSDME to GSDME N-terminal [20]. Western blot analysis of Bax, Bcl2, cleaved caspase-3, and N-terminal GSDME was performed, and caspase-3 activity was detected by Caspase 3 Activity Assay Kit. As shown in Fig. 4e, the expression of Bax, a proapoptotic factor being able to promote caspase-3, was upregulated by metal-gallate nanoparticles, whereas the expression of Bcl-2, an antiapoptotic protein being able to suppress caspase-3 activity was downregulated. As a consequence, the cleaved caspase-3 and N-terminal GSDME were upregulated, which is a typical feature of pyroptosis. It was apparent that the expression of pro-pyroptotic proteins were up-regulated by metal-gallate nanoparticles, following an order of MnGA > FeGA > CaGA. Caspase 3 activity in the cells with metal-gallate nanoparticles treatment was also higher than that of GA group, especially the FeGA and MnGA groups (Fig. 4f). The above results indicate that the metal-gallate nanoparticles were superior to the free GA in enhancing pyroptosis of MG-63 cells, which was likely ascribed to synergistic actions of metal ions. As shown in Fig. 4a, the metal-gallate nanoparticles enhanced the generation of intracellular ROS. ROS have been reported to activate Bax through a Bcl-2-suppressible pathway and thus activate the caspase3 pathway [40, 41]. The canonical caspase-1 inflammasome can induce pyroptosis, or caspases-4, -5, and -11 bound to lipopolysaccharide can directly induce pyroptosis, however, pyroptosis is also controlled by caspase-3/GSDME as reported in recent studies [42]. Zhou et al. [20] reported that Fe ions produced ROS through Fenton reactions and activated caspase3-GSDME, which in consequence induced pyroptosis. Therefore, it is probably that the metal-gallate nanoparticles firstly up-regulated Bax and thus activated caspase-3 by the upregulated intracellular ROS, and in turn GSDME was then cleaved by active caspase-3 at its middle linker, liberating the gasdermin-N domain and forming pores on cell membrane triggering pyroptosis. In this study, a certain degree of pyroptosis was found in the cells treated with the free GA. However, GA-induced pyroptosis has not been reported in the literatures available. It was found that the concentrations of GA and the treatment duration they used were lower and shorter, compared to those used in the present study, which possibly accounts for our findings in the different role of GA.

Since Ca ions don’t have the ability to induce Fenton reactions, however, the intracellular ROS generated by CaGA was comparable to those caused by Fe and Mn ions, and higher than that for the GA group. Therefore, it is likely indicated that Ca ions synergized with GA in a different unknown way. Tan and Huang et al. [43–45] reported that once the functions of the endoplasmic reticulum and mitochondria was impaired by drugs, the subsequent intracellular enrichment of Ca ions could upregulate the ROS generation. Therefore, for the CaGA nanoparticle, it is hypothesized that GA first resulted in the dysfunction of endoplasmic reticulum and mitochondria upon CaGA are uptaken and decomposed, and the introduced Ca ions lead to the overload of intracellular Ca (II) ion, which ultimately enhanced ROS generation. The relevant underlying mechanisms will be further explored in our future work.

### MnGA enhanced mild-temperature PTT by down-regulating HSP via exhausting ATP

**Fig. 5:**
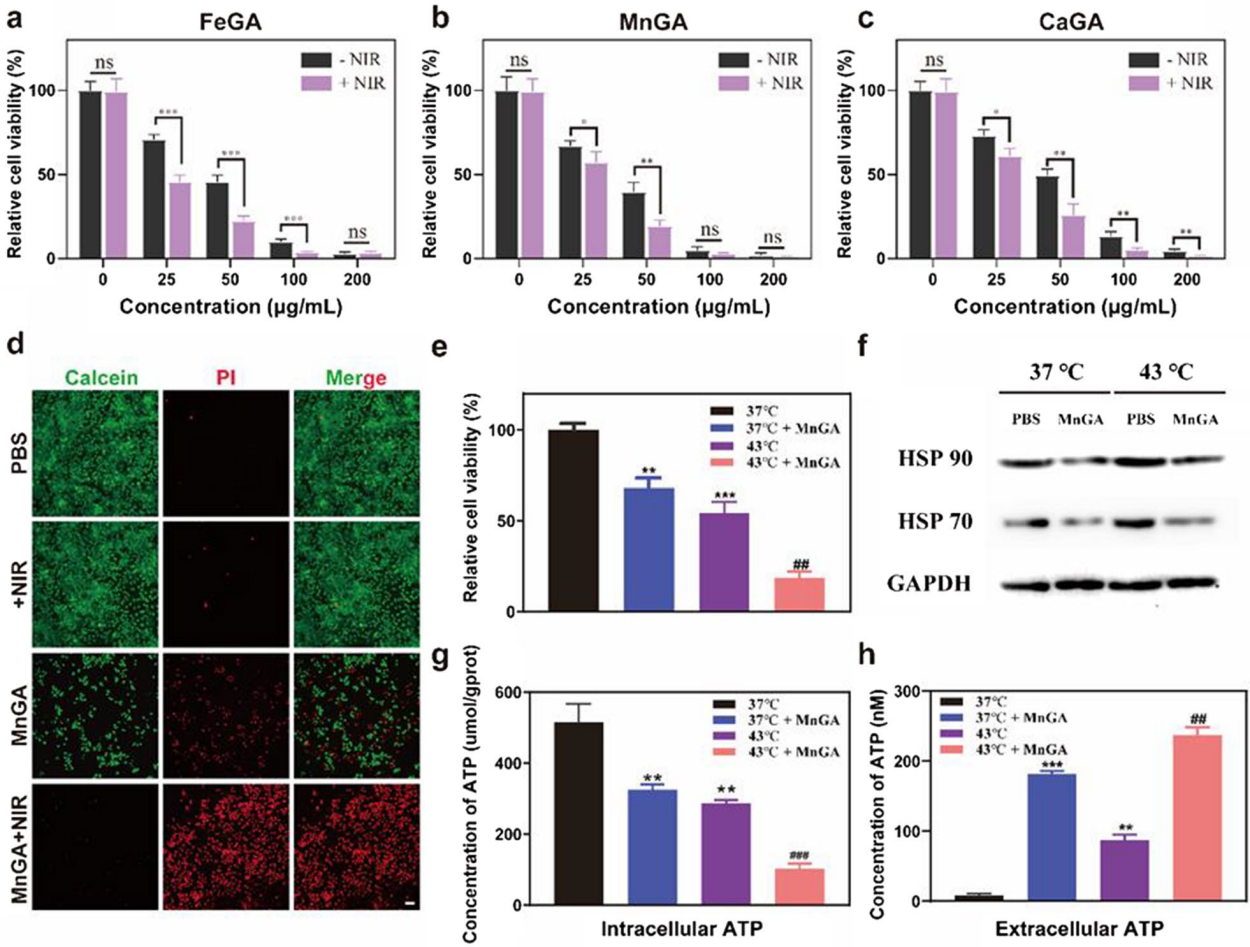
MnGA nanoparticles enhance PTT by down-regulating HSP via exhausting ATP. **a**–**c** Cell viabilities of MG-63 cells co-incubated with FeGA, MnGA and CaGA nanoparticles for 6 h at different concentrations with and without irradiation respectively (1.5 W/cm^2^, 10 min). **d** Live/dead images of MG-63 cells (stained by calcein and PI) treated with MnGA nanoparticles and PBS on exposure to NIR irradiation or not. Scale bars: 200 μm. **e** Relative cell viabilities when coincubated with MnGA nanoparticles (15 μg/ml) or PBS for 12 h in 37 or 43 °C. **f** Intracellular HSP70 and HSP90 protein expression in MG-63 cells in above groups. **g** Concentrations of intracellular ATP detecting by an ATP Determination Kit. **h** ATP secretion in the cell medium after various treatments detecting by an ATP Determination Kit

Given that tumor cells are less heat tolerant than normal cells [46], and hyperthermia could accelerate the Fenton reaction [47], by using PTT and CDT together is perceived as a promising strategy for enhanced anticancer efficacy. From this aspect, the metal-gallate nanoparticles showed high potential in realizing the combinatory CDT/PTT due to their photothermal property and ability to induce intracellular ROS and pyroptosis. To validate this hypothesis, cytotoxicity experiments were conducted by treating MG-63 human osteosarcoma cells with metal-gallate nanoparticles of multiple concentrations (0, 25, 50, 75, 100 μg/ml) with or without NIR irradiation. In comparison with the control group, Fig. 5a–c showed that metal-gallate nanoparticles groups had a better ability to destroy tumor cells under 808 nm laser irradiation. MnGA nanoparticles were opted for further in vitro and in vivo experiments because of their strong anti-cancer ability and applicable photothermal performance. Additional file 1: Figure S12 showed that MnGA could be up-taken by the tumor cells, the amount of the up-taken nanoparticles increased as the co-cultivation time was prolonged. Figure 5d showed the images of calcein-AM/propidium iodide (PI) stained MG-63 cells treated with PBS and MnGA nanoparticles with and without NIR irradiation. It can be obviously seen that the MnGA nanoparticle itself induced massive cell death, indicating its promising chemotherapeutic potential. NIR irradiation (10 min) increasing the temperature to 43 °C, almost killed all the cells fed with the MnGA nanoparticles, but rendered no harm to the cells treated with PBS, indicating the potent combinational anticancer effects of CDT and PTT of the MnGA nanoparticle.

In traditional PTT, hyperthermia usually with a temperature higher than 50 °C could effectively eliminate tumor cells whereas simultaneously leaving damages to normal tissues [48]. Hence mild temperature (≤ 45 °C) PTT was proposed to be a promising anti-tumor therapy due to its biosafety and biocompatibility [49]. The above results pointed out the possibility of MnGA in the mild-temperature thermal therapy (43 °C). To further validate that the enhanced cytotoxicity was caused by the heat generated from NIR irradiation on the MnGA, MG-63 cells treated with PBS and MnGA were incubated at 37 °C and 43 °C for 8 h. As can be seen from Fig. 5e, significantly enhanced reduction in cell viability was found for the group of MnGA + 43 °C, indicating the contribution of heat to the anti-cancer effect. Previous study reported that damage of cell like apoptosis caused by moderate-temperature thermal therapy (e.g., 45 °C) can be avoided or weakened by the repairing mechanism of cells through overexpression of heat shock proteins (HSP) [50]. To further understand the underlying synergistic anti-tumor mechanism of the MnGA, the two main HSP proteins (HSP70 and HSP90) expressions were studied. HSPs expression has been reported to be dependent on ATP consumption [51], hence mitochondrial dysfunction-induced ATP underproduction can down-regulate the expression of HSP. Besides, ATP was reported to be able to be released to extracellular matrix (ECM) through the pores on pyroptosis cell, and we have proved that MnGA induced pyroptosis through caspase-3-GSDME pathway. Therefore, it is of great necessity to shed light into how ATP production was regulated by MnGA and incubation at evaluated temperatures. As shown in Fig. 5g, both 43 °C incubation and MnGA treatment suppressed the intracellular ATP generation. Besides, MnGA also induced massive ATP efflux into extracellular matrix (Fig. 5h). Therefore, we believe that the inhibition of ATP generation and the ATP efflux synergistically contributed to the downregulation of HSPs, eventually enhanced the mild-temperature PTT of the MnGA. Collectively, above results indicated that by exhausting intracellular ATP via downregulation of HSPs, MnGA could be an effective adjuvant for mild-temperature PTT.

### In vivo photothermal image and anticancer evaluation

**Fig. 6:**
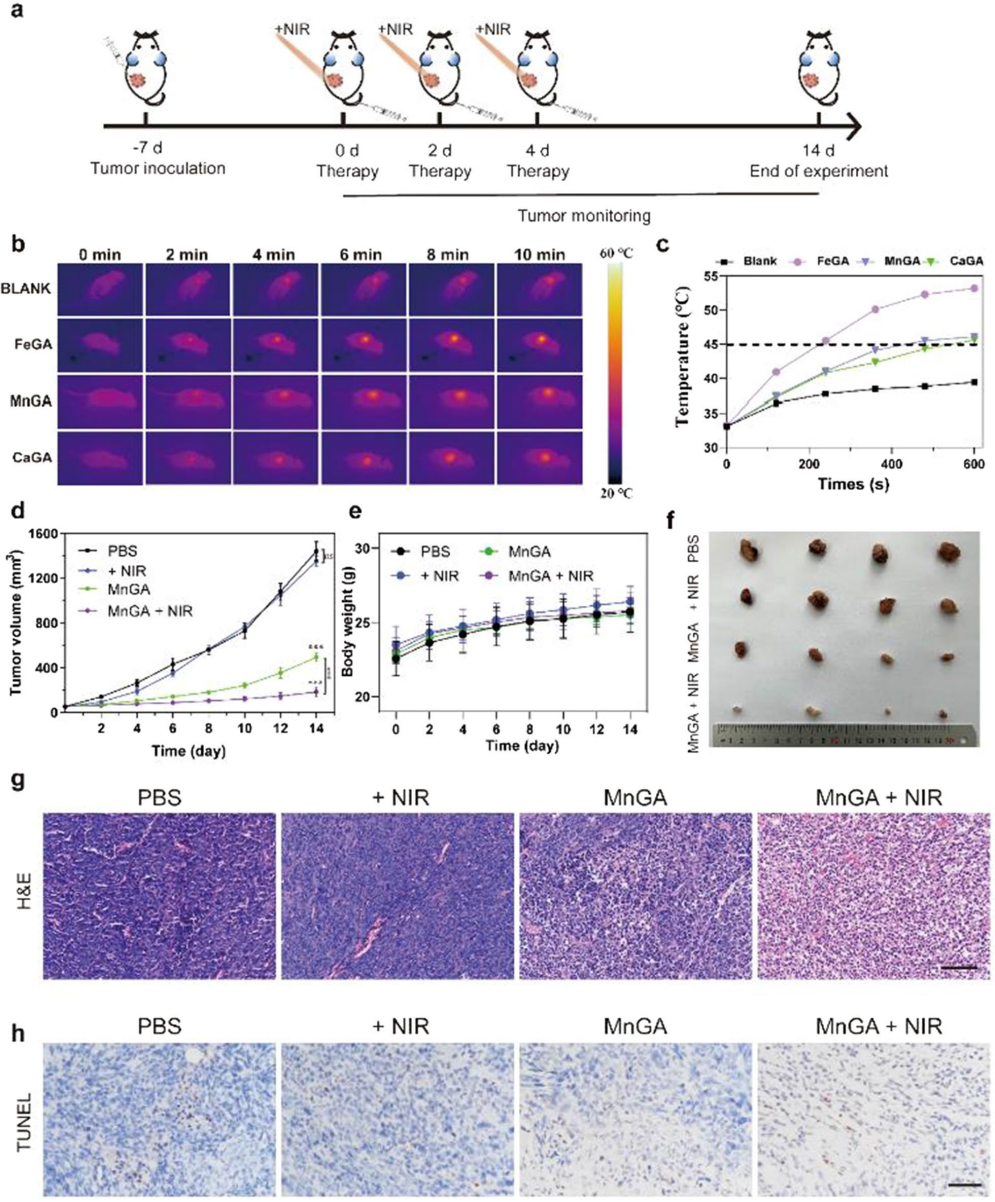
In vivo photothermal images and antitumor effect evaluation of MnGA-based mild-temperature PTT. **a** Schematic diagram of the in-vivo experiment to evaluate the anti-tumor effect of MnGA nanoparticles. **b** The representative thermal images of the mice with intravenous administration of PBS, FeGA, MnGA and CaGA (10 mg/kg) under NIR irradiation for 10 min (1.5 W/cm^2^). **c** The corresponding temperature changes over time curve of tumor regions with injection of PBS, FeGA, MnGA and CaGA. **d** Tumor volume growth curves of each group (n = 4). **e** The body weight changes in the four groups of mice. **f** The images of the tumors on day 14 after section. **g** H&E staining of tumor sections in four different groups. **h** TUNEL analysis of the tumor sections from various groups on the 14th day of the experiments. Scale bars: **g**, **h**) 100 μm. ns: no significance, ***p < 0.001

As shown in Fig. 6a, MG-63 osteosarcoma cells were used to create a mouse subcutaneous tumor model. Briefly, the MG-63 tumor-bearing mice having tumors volume of about 100 mm^3^ were grouped and treated with PBS, + NIR, metal-gallate nanoparticles, metal-gallate nanoparticles plus NIR laser (1.5 W/cm^2^, 10 min). The NIR irradiation was performed every two days (day 0, 2 and 4). Before the systematic animal experiment, the in vivo photothermal effect of FeGA, MnGA and CaGA were assessed using a thermal imager with different irradiation duration. As shown in Fig. 6b and Fig. 6c, the temperature rose to 54 °C for the FeGA group after NIR irradiation for 10 min, whereas the temperature reached only to 45 °C for MnGA and CaGA. As we mentioned before, high temperature ablation of tumors leads to several heating damage of normal tissues around the tumor [13, 52]. Therefore, the irradiation duration (10 min) corresponding to the temperature of 43 °C was selected for the animal studies. Although the photothermal conversion efficiency of FeGA was the highest, the MnGA was superior and selected for the animal studies due to its potent chemotherapeutic effects.

Figure 6d shows the tumor growth curves from different groups. It can be seen that the NIR group showed negligible tumor inhibition, while tumors were inhibited to some extent by the MnGA group. Significant tumor suppression was observed in the MnGA + NIR group. The body weight of the mouse for the MnGA and MnGA + NIR groups did not show significant difference from the control group (Fig. 6e), indicating the good biocompatibility of MnGA nanoparticles. The images of the primary tumors and their weights were displayed in Fig. 6f and Additional file 1: Figure S13, showing a similar trend to that observed in Fig. 6d. Staining with hematoxylin and eosin (H&E) results showed that combined therapy significantly reduced tumor cell damage, characterized by shrinkage and nuclear condensation of the tumor cells (Fig. 6g). Terminal deoxynucleotidyl transferase dUTP nick end labeling (TUNEL) assay illustrated that the MnGA + NIR group showed the highest mortality rate of tumor cells (Fig. 6h). Since the in vitro experiments proved that MnGA promoted cell death by induction of pyroptosis besides apoptosis, and the enhanced PTT was also related to ATP exhaustion via pyroptosis, we further confirmed that the activation of pyroptosis by MnGA was via Caspase-3-GSDME pathway in vivo. As seen in Additional file 1: Figure S14, caspase-3 and GSDME-N were up-regulated in MnGA and MnGA plus NIR group, especially in the latter group. In summary, the animal studies proved that the MnGA with the NIR irradiation could significantly eliminate tumor ascribed to the combination of the potent chemotherapy and mild-temperature PTT.

### In vivo biodistribution and biosafety evaluation of MnGA

**Fig. 7:**
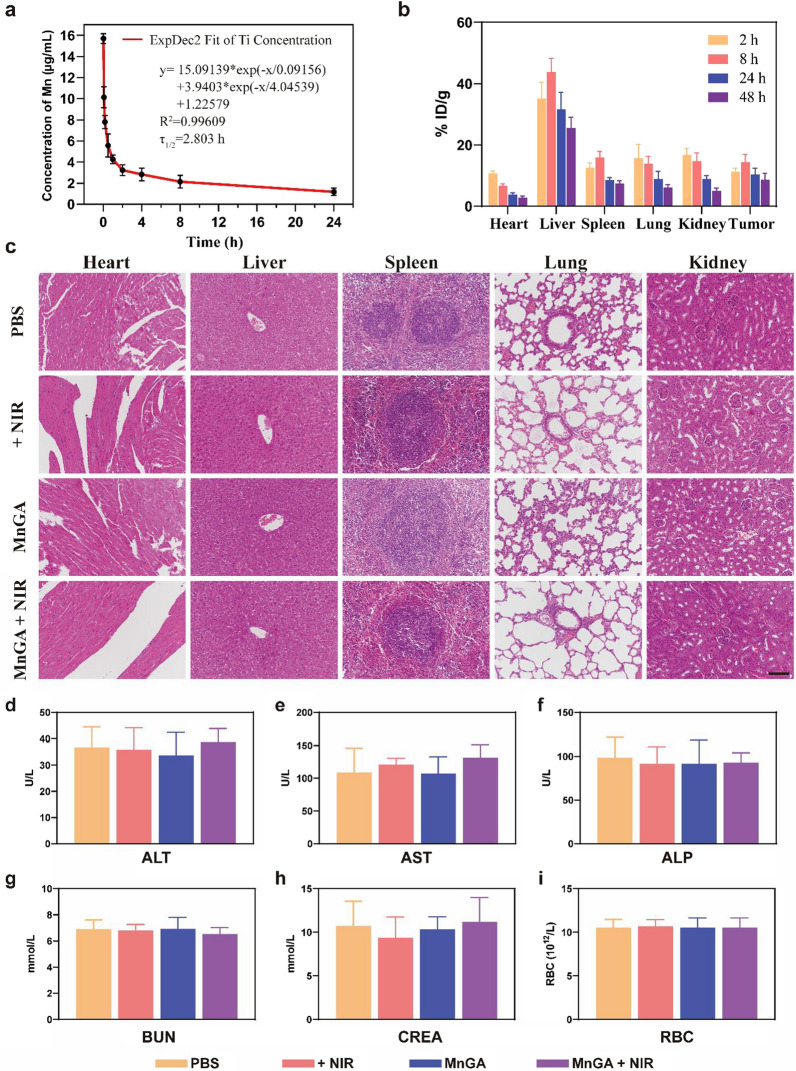
In vivo biodistribution and biosafety evaluation of MnGA Nanoparticles. **a** Blood-circulation curves of MnGA nanoparticles administrated intravenously (10 mg/kg, n = 4). The half-life period (τ1/2) of Mn was calculated to approximate 2.803 h. **b** Biodistributions of MnGA nanoparticles (%ID/g of Mn) in tumors, heart, liver, spleen, lung and kidney after intravenous administration (2, 8, 24 and 48 h, n = 4) **c** H&E staining of main organs such as the heart, liver, spleen, lung and kidney for biosafety evaluation of four groups in vivo. **d**–**i** Blood biochemical profile and blood routine tests on the 14th day in groups including Blank, NIR, MnGA and MnGA + NIR. Scale bar: **c** 100 μm

As shown in Fig. 7, in vivo biodistribution and biosafety were assessed to test toxicity or side effects induced by MnGA or PTT treatment. Firstly, by using inductively coupled plasma mass spectrometry (ICP-MS), the pharmacokinetics of MnGA were evaluated in vivo. Observing the blood-circulation curves of Mn element, it can be seen that the MnGA was removed from blood vessels gradually, and the half-life periods (τ1/2) in vivo were about 2.8 h (Fig. 7a). On the basis of the typical EPR effect in solid tumors, MnGA was enriched at tumor sites with a targeting efficiency of 14.39% ID/g at 8 h (Fig. 7b).

Histological analysis was performed at the end of the treatment. As can be seen from the H&E sections (Fig. 7c), main organs including heart, liver, spleen, lung, and kidney did not show any obvious tissue damage. Besides, the blood was also tested for blood biochemistry and routine tests. Figure 7d–f showed that there was no significant increase in glutamic-pyruvic transaminase (ALT), glutamic oxalacetic transaminase (AST) and alkaline phosphatase (ALP). Likewise, the blood urea nitrogen (BUN) and creatinine (CREA) levels were in the normal range (Fig. 7g and h), indicating no significant damage to the kidneys. In each group, the concentration of main blood cells such as red blood cells (RBC) was not significantly difference from that of the control group(Fig. 7i). In general, these results all together proved the biosafety of the developed MnGA nanoparticles, thus, they have the potential to be clinically useful in the future.

## Conclusions

Herein, we constructed a metal-gallate nanoparticles for exhausting cellular ATP by both inhibiting mitochondrial ATP generation and initiating pyroptosis accompanied by ATP efflux, which greatly inhibits HSPs and achieves mild PTT with superior tumor therapeutic effect. The proposed metal-gallate nanoparticles, displaying efficient clearance of tumors both in vitro and in vivo, mainly attained the following progresses: (1) Nanoformulation of GA promoted the stability and bioavailability of GA while retaining its antineoplastic activities; (2) Besides the already known apoptosis pathway, metal-gallate nanoparticles also triggered pyroptosis via caspase-3/GSDME pathway with the synergistic actions of metal ions. (3) The MnGA nanoformulation could downregulate HSP via exhausting ATP, eventually enhancing mild PTT effect. Thanks to the photothermal effect of nanoformulation, the MnGA nanoparticles under NIR laser irradiation effectively killed OS with no obvious side effects. Our results strongly indicate the highly promising therapeutic prospects of an efficient and safe anti-tumor strategy based on mild PTT against OS in synergy with mitochondrial destruction-mediated pyroptotic induction.

## Supplementary Information

** Additional file 1**: Supplementary documents.

## Data Availability

The data that support the findings of this study are available from the corresponding authors upon reasonable.

## Abbreviations

GA: Gallic acid
ROS: Reactive oxygen species
OS: Osteosarcoma
PTT: Photothermal therapy
HSPs: Heat shock proteins
ATP: Adenosine triphosphate
DMEM: Dulbecco’s Modified Eagle Medium
CCK-8: Cell counting kit-8
FTIR: Fourier transform infrared spectroscopy
ICP: Inductively coupled plasma-optical emission spectrometry
ALT: Glutamic-pyruvic transaminase
AST: Glutamic oxalacetic transaminase
ALP: Alkaline phosphatase
BUN: Blood urea nitrogen
CREA: Creatinine
