# Supplementary material for: Enhanced mild-temperature photothermal therapy by pyroptosis-boosted ATP deprivation with biodegradable nanoformulation
Source: J Nanobiotechnology. 2023 Feb 23;21:64. doi: 10.1186/s12951-023-01818-1 (PMC9948333; doi:10.1186/s12951-023-01818-1)
Supplement: Supplementary file 1 — Additional file 1: Supplementary documents. [file 12951_2023_1818_MOESM1_ESM.docx]

**Supporting Information**

**Enhanced Mild-Temperature Photothermal Therapy by Pyroptosis-Boosted ATP Deprivation with** **Biodegradable Nanoformulation**

Kaiyuan Liu^b^†, Li Zhang^b^†, Hengli Lu^b^†, Yingfei Wen^a^, Bo Bi^a^, Guocheng Wang^c^, Yingying Jiang^d*^, Leli Zeng^a*^, Jing Zhao^a, b*^

^a^ Scientific Research Center, The Seventh Affiliated Hospital of Sun Yat-sen University, Shenzhen 518107, P. R. China

^b^ School of Medicine, Tongji University, Shanghai 200072, P. R. China.

^c^ Research Center for Human Tissues and Organs Degeneration, Shenzhen Institute of Advanced Technology, Chinese Academy of Science, Shenzhen, Guangdong 518055, China.

^d^ Institute of Translational Medicine, Shanghai University, Shanghai 200444, P. R. China

† Kaiyuan Liu, Li Zhang and Hengli Lu have contributed equally to this work and share first authorship.

*corresponding author:

E-mail: yjiang8@shu.edu.cn (Jiang Y)

E-mail: zenglli6@mail.sysu.edu.cn (Zeng L)

E-mail: zhaoj265@mail.sysu.edu.cn (Zhao J)

**Supplementary figures**

­­
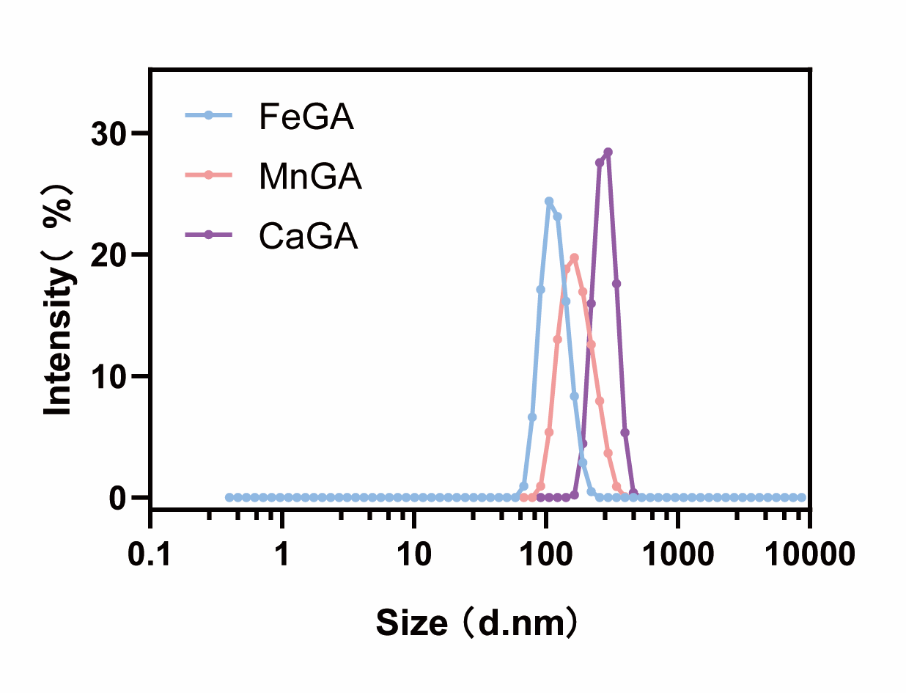


**Figure S1.** Hydrodynamic diameter distribution of FeGA, MnGA and CaGA.


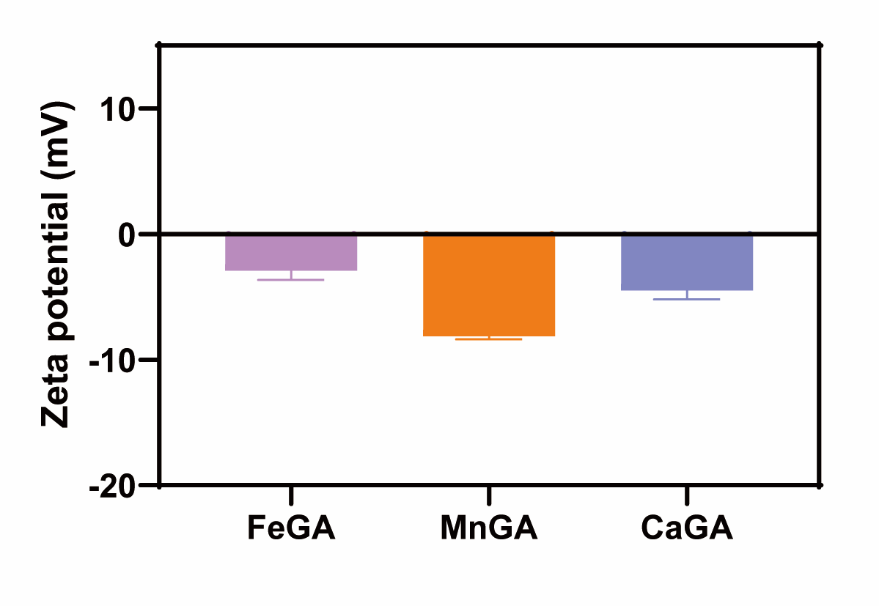


**Figure S2.** Zeta potentials of FeGA, MnGA and CaGA.


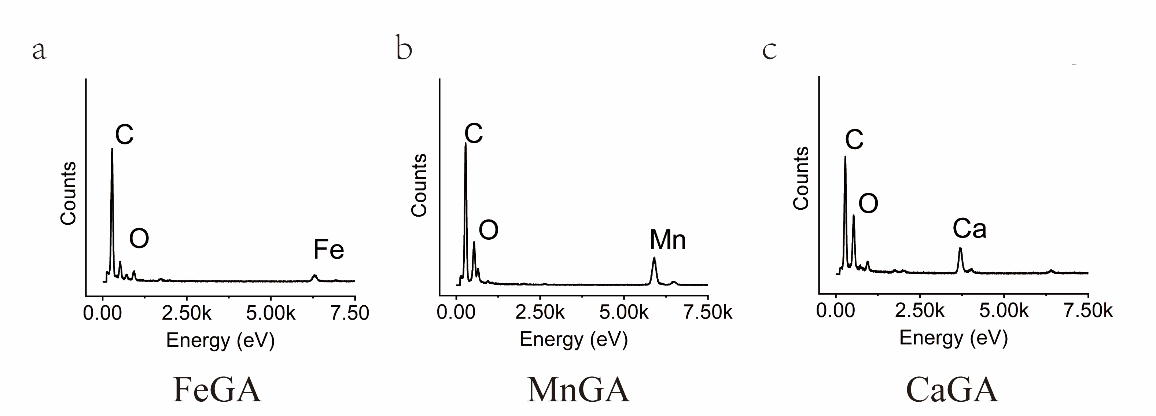


**Figure S3.** X-ray energy dispersive spectroscopy (EDS) of FeGA, MnGA and CaGA nanoparticles indicating the coexistence of Fe, Mn, Ca elements respectively.

**
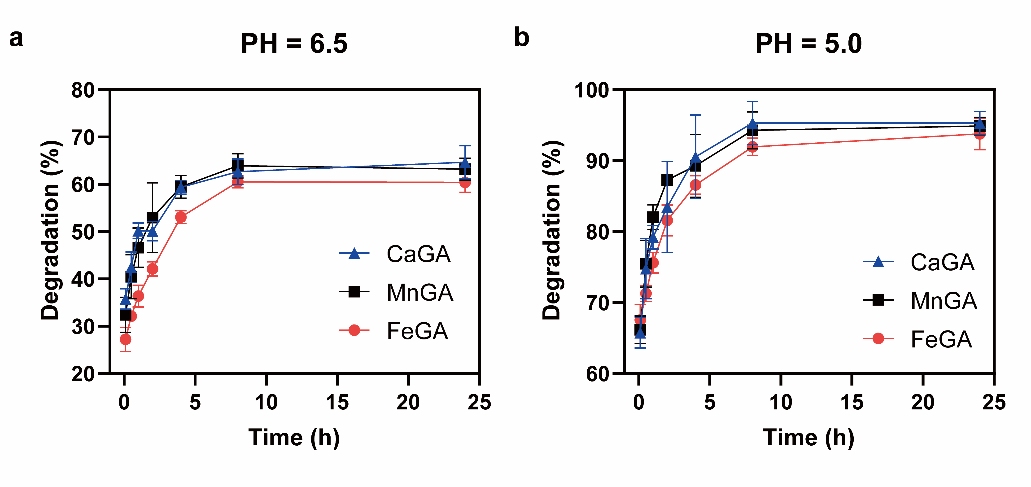
**

**Figure S4.** Degradations of FeGA, MnGA and CaGA nanoparticles in PBS at pH 6.5 (a) and 5.0 (b).


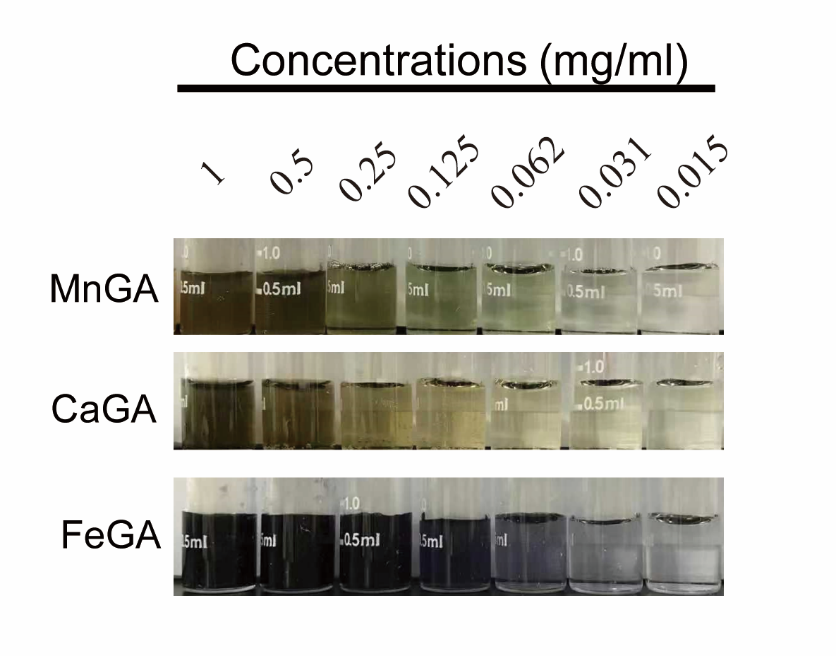


**Figure S5.** Photographs of the FeGA, MnGA and CaGA of different concentrations.


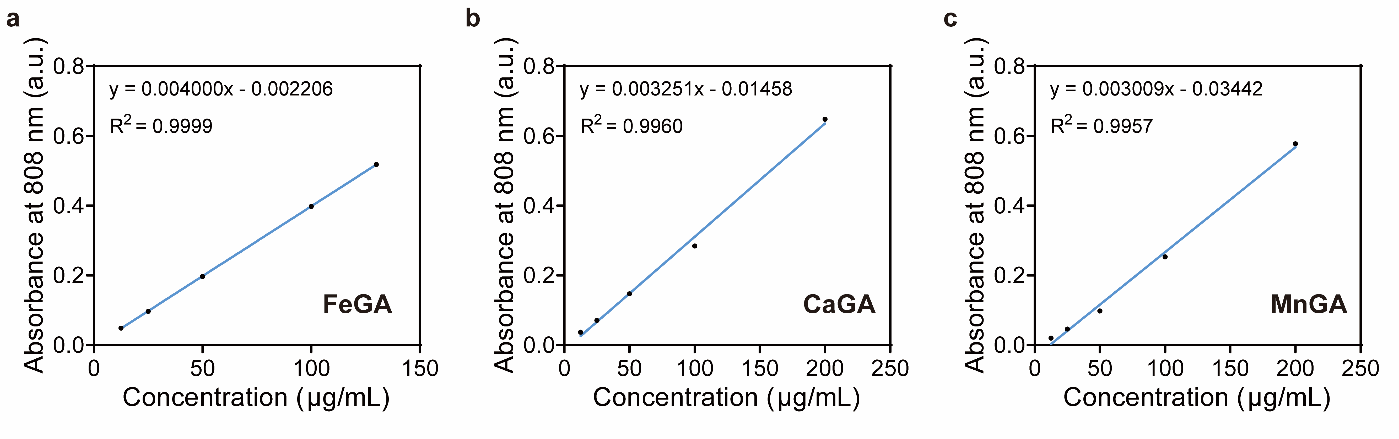


**Figure S6.** Normalized light absorbance at fixed wavelength (λ = 808 nm) over the length of cell (A/L) at elevated concentrations. (a) FeGA, (b) CaGA and (c) MnGA.


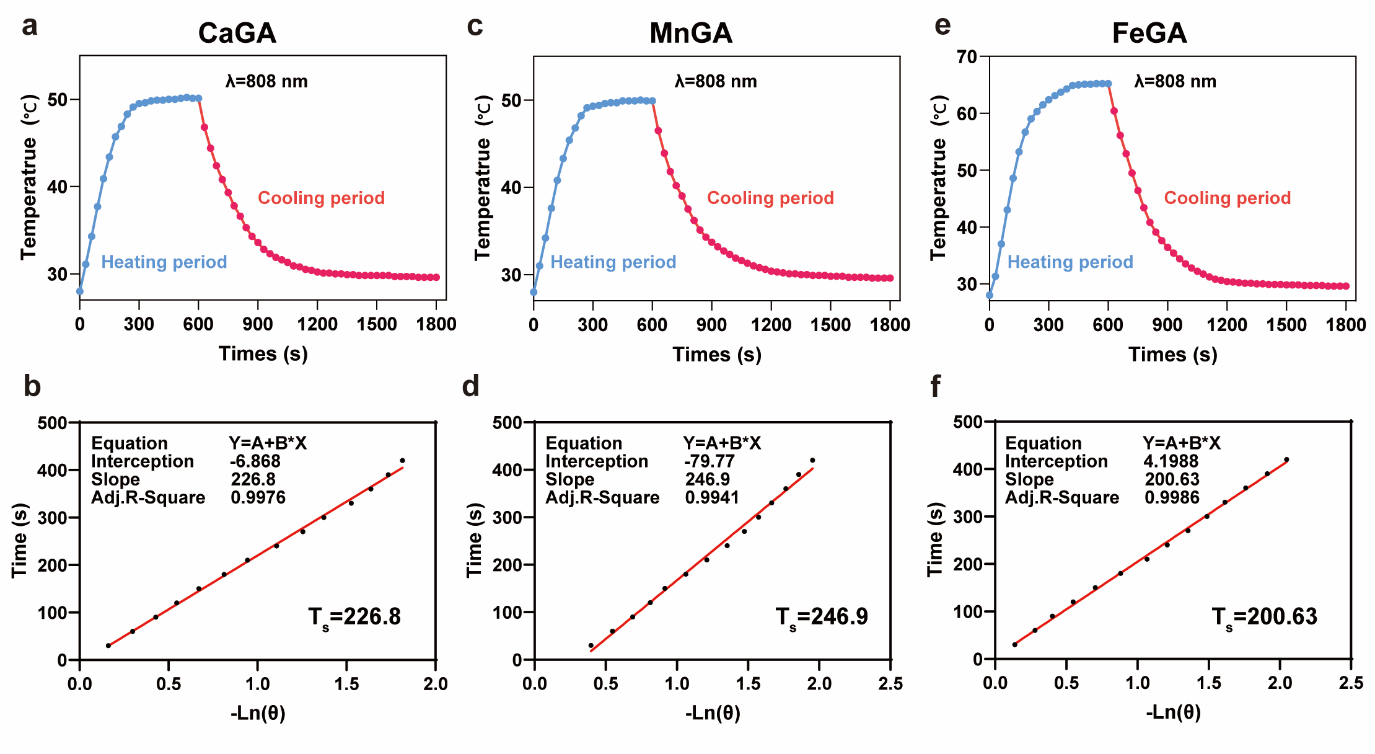


Figure S7. The photothermal-conversion efficiencies (η) of the metal-gallate nanoparticles. The temperature changes of CaGA (a), MnGA (c) and FeGA (e) over laser irradiation on/off cycles at a constant concentration of 400 μg/ml under the irradiation (2 W cm-2). Heat transferring time constant of CaGA (b), MnGA (d) and FeGA (f) was determined by using the linear time data from the cooling period versus negative natural logarithm of driving force temperature, which was obtained from the cooling stage.


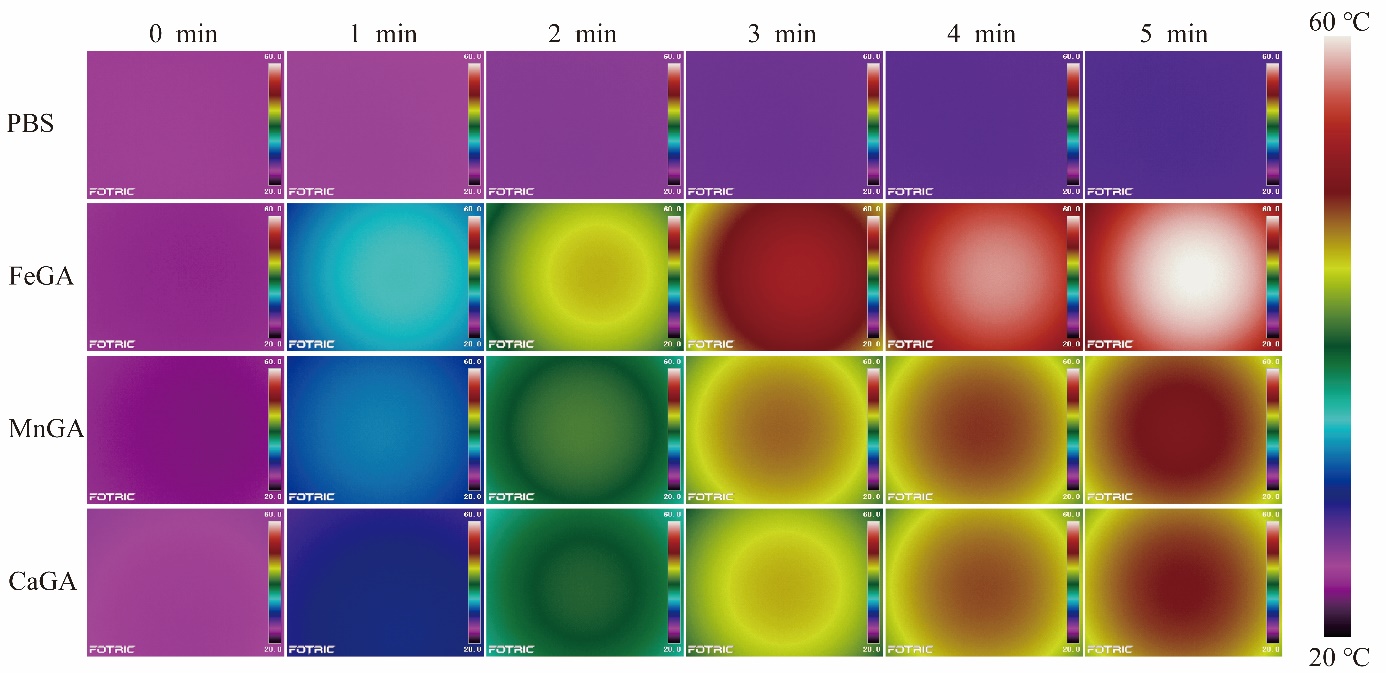


**Figure S8.** Infrared thermal images of PBS, FeGA, MnGA and CaGA exposed to 808 nm laser for 5 min (400 μg/ml).


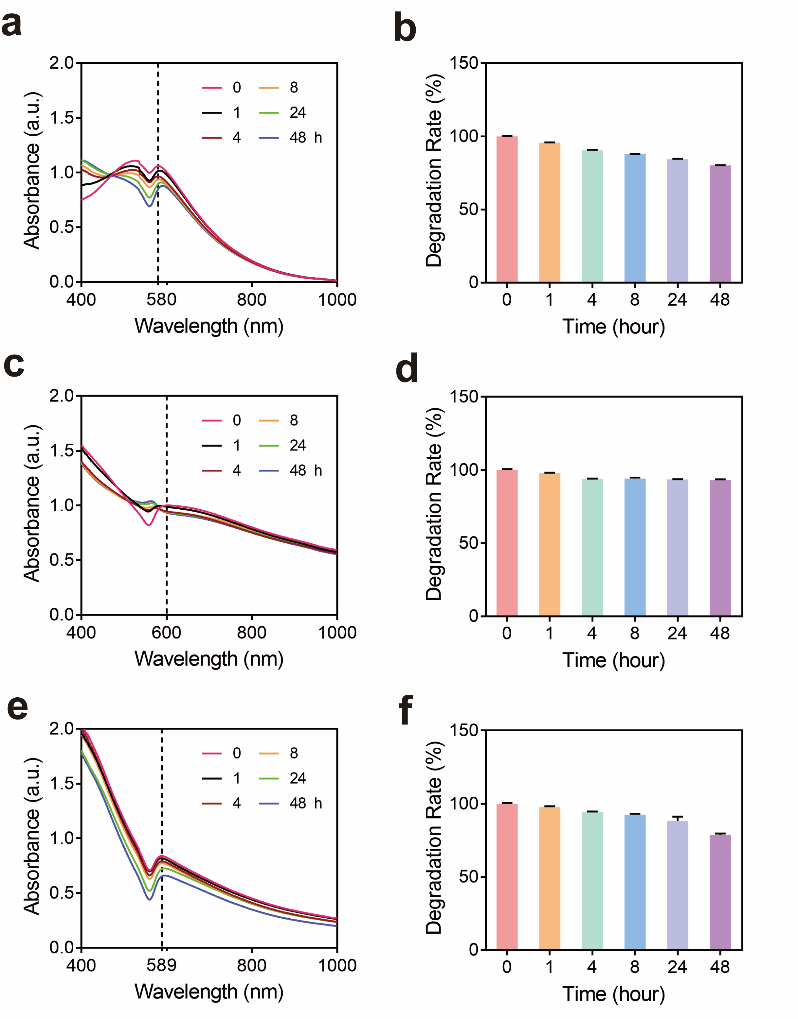


**Figure S9.** NIR absorption variation of FeGA (a), CaGA (c) and MnGA (e) in DMEM over 48 hours and corresponding degradation rate (b, d, f).


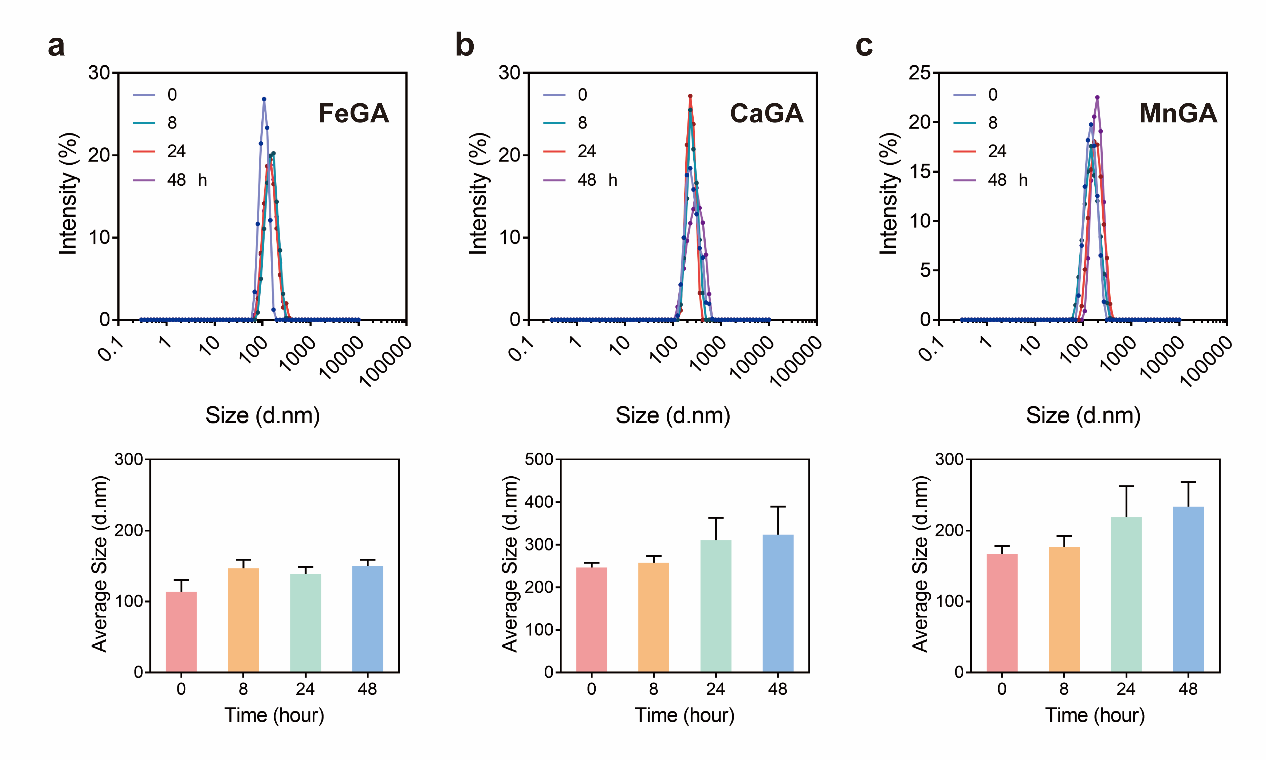


**Figure S10.** DLS variation of FeGA (a), CaGA (b) and MnGA (c) in DMEM over 48 hours


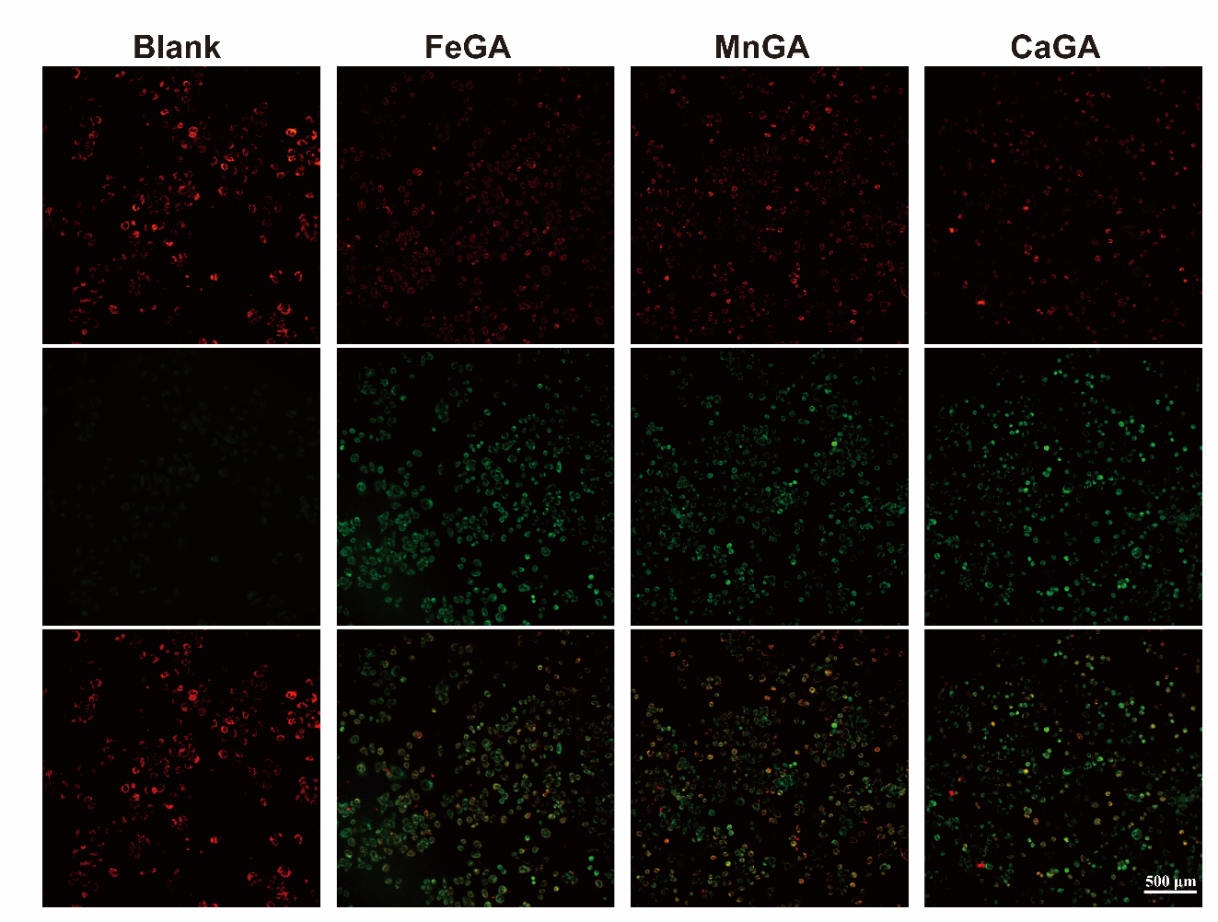


**Figure S11**. Fluorescence microscopy images of JC-1-labeled MG-63 cells treated with metal-gallate nanoparticles at 37 °C for 24 h

**
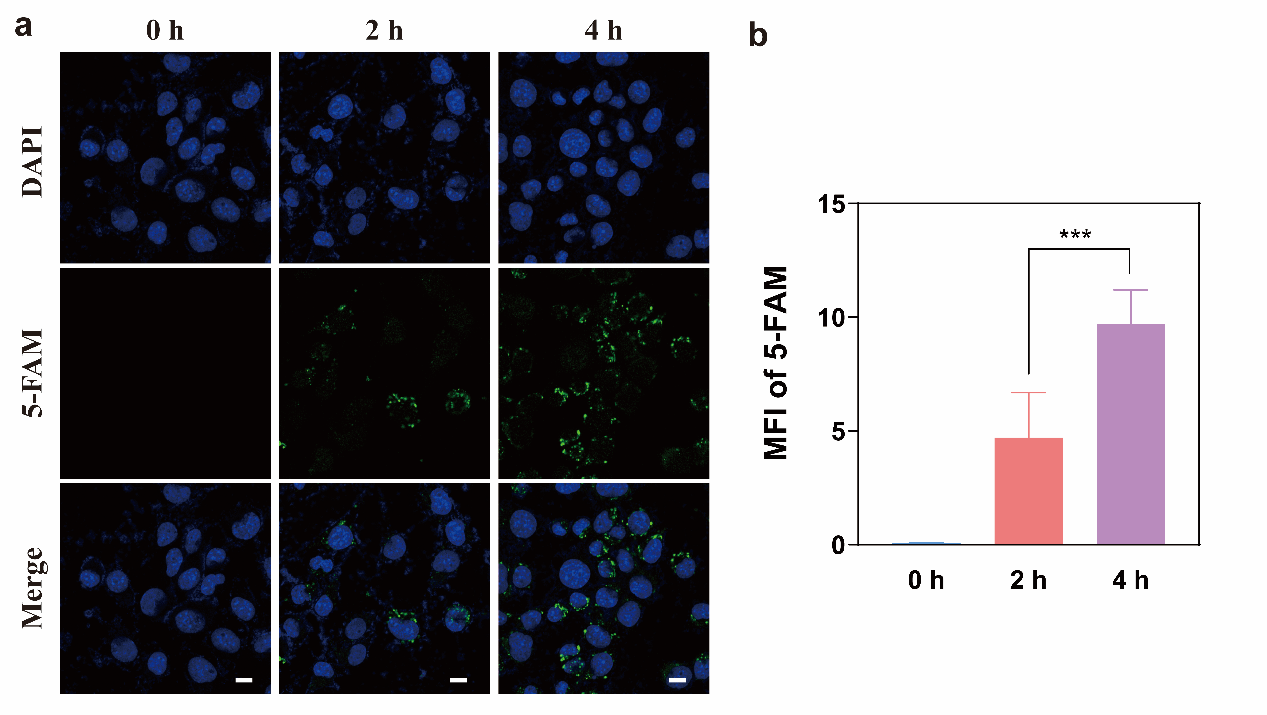
**

**Figure S12.** *In vitro* cellular uptake assessment. MnGA-5FAM were incubated with MG-63 cells, and after incubating for 2 h and 4 h, cell nucleus were stained with DAPI, and were observed by fluorescence microscope (a) and corresponding quantitative [fluorescence intensity](javascript:;) of 5-FAM (b). Scale bars: 10 μm


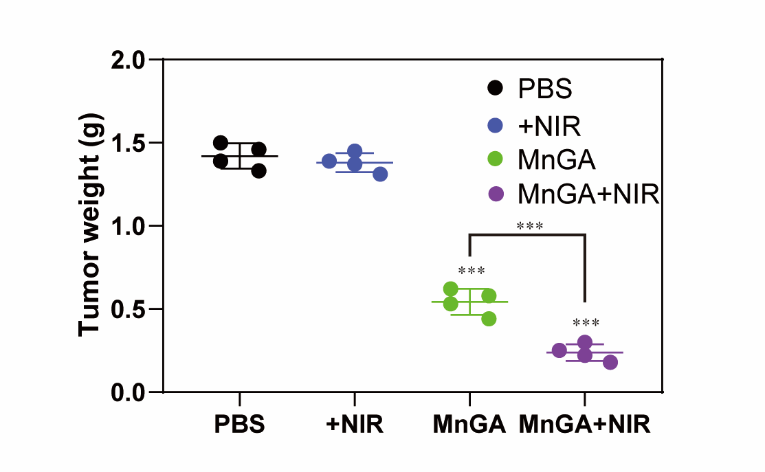


**Figure S13.** Tumor weight of tumors after section from different groups 14 days after the first treatment.


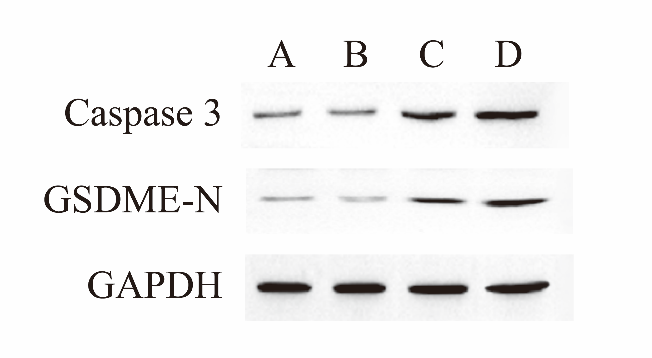


**Figure S14.** Western blot assay of cleaved caspase-3 and N-terminal gasdermin E (N-GSDME) expression of tumor tissue in groups including PBS, NIR, MnGA, and MnGA + NIR
